# Single-cell RNA-sequencing identifies disease-associated oligodendrocytes in male APP NL-G-F and 5XFAD mice

**DOI:** 10.1038/s41467-023-36519-8

**Published:** 2023-02-13

**Authors:** Hanseul Park, Byounggook Cho, Hongwon Kim, Takashi Saito, Takaomi C. Saido, Kyoung-Jae Won, Jongpil Kim

**Affiliations:** 1 Department of Chemistry, Dongguk University, Seoul, 100‐715 Republic of Korea; 2 Laboratory for Proteolytic Neuroscience, RIKEN Center for Brain Science, Wako-shi, Saitama, Saitama Japan; 3 Department of Neurocognitive Science, Institute of Brain Science, Nagoya City University Graduate School of Medical Sciences, Nagoya, Aichi Japan; 4 Department of Computational Biomedicine, Cedars-Sinai Medical Center 700 N, San Vicente Blvd., West Hollywood, CA USA

**Keywords:** Alzheimer's disease, Oligodendrocyte

## Abstract

Alzheimer’s disease (AD) is associated with progressive neuronal degeneration as amyloid-beta (Aβ) and tau proteins accumulate in the brain. Glial cells were recently reported to play an important role in the development of AD. However, little is known about the role of oligodendrocytes in AD pathogenesis. Here, we describe a disease-associated subpopulation of oligodendrocytes that is present during progression of AD-like pathology in the male App^NL-G-F^ and male 5xFAD AD mouse brains and in postmortem AD human brains using single-cell RNA sequencing analysis. Aberrant Erk1/2 signaling was found to be associated with the activation of disease-associated oligodendrocytes (DAOs) in male App^NL-G-F^ mouse brains. Notably, inhibition of Erk1/2 signaling in DAOs rescued impaired axonal myelination and ameliorated Aβ-associated pathologies and cognitive decline in the male App^NL-G-F^ AD mouse model.

## Abstract (web summary)

Oligodendrocytes have been increasingly shown to be involved in Alzheimer’s disease (AD). Here, the authors perform single-cell RNA-sequencing on APP NL-G-F mice and describe a disease-associated oligodendrocyte (DAO) population. They find inhibition of Erk1/2 signaling in DAOs rescues impaired axonal myelination and cognitive decline in an AD mouse model.

## Introduction

AD is one of the most common forms of dementia and is characterized by progressive loss of memory and cognitive functions, however, the etiology of this disease remains largely unknown^1^. AD brains have several distinctive neuropathological features including intracellular neurofibrillary tangles that consist primarily of abnormally phosphorylated tau proteins and senile plaques whose main components are amyloid beta (Aβ) peptides^2,3^. This disease progresses through a significant reduction of synapses coupled with neuroinflammation^4^. Numerous studies have linked non-neuronal glial cells such as microglia or astrocytes to AD pathogenesis^5–7^. For example, many studies have reported that microglia respond to disease conditions and mediate disease progression^8,9^. Particularly, a recent single-cell analysis demonstrated the activation of a unique disease-associated microglia (DAM) subtype in AD mice and postmortem AD brains^10^. Moreover, astrocytes have also been linked to AD pathogenesis. A recent study also reported a previously undescribed subpopulation of disease-associated astrocytes (DAAs)^11^. However, several cell types associated with oligodendrocytes during AD progression remain uncharacterized.

Recent evidence suggests that demyelination is an important pathophysiological feature in AD^12,13^. Due to the role of oligodendrocytes in the myelination process, AD pathogenesis could potentially be ascribed to oligodendrocyte alteration or dysfunction. Consistent with this idea, some studies have reported that inhibition of oligodendrocyte differentiation in the mouse brain impairs learning and cognitive functions. An increase in immature or abnormal oligodendrocytes has also been observed in mouse models of AD^14–16^. Indeed, recent studies have reported that Aβ exerts toxic effects on oligodendrocyte precursor cells and/or oligodendrocytes in the AD brain^17,18^. Interestingly, a previous single-cell transcriptomic analysis of post-mortem human brain samples found that myelination-related genes were dysregulated in several cell types including oligodendrocytes and oligodendrocyte precursor cells, suggesting that myelination and demyelination may be important pathophysiological drivers of AD progression. A recent single-cell analysis elucidated disease-related perturbations of myelination-associated genes in oligodendrocytes and their precursor cells in postmortem AD brains^19^. Furthermore, in the 5xFAD model, oligodendrocytes manifested disease-associated gene signatures that contribute to impaired axonal myelination. Therefore, these studies suggest that oligodendrocyte dysfunction may be closely associated with AD progression.

Two recent studies reported the disease-associated oligodendrocytes in AD mouse models and postmortem AD brain tissues^20,21^. While these studies identified the subpopulation of oligodendrocytes and characterized their molecular signatures responses in disease states, an understanding of transcriptomic pseudotime trajectory toward the disease-associated oligodendrocytes in AD progression is lacking. More importantly, it is unclear the specific mechanisms that mediate oligodendrocyte impairment in AD progression and the therapeutic potentials of these populations against AD progression. Thus, the systematic understanding of the mechanisms that develop disease-associated oligodendrocytes during disease progression may advance therapies for AD.

Our study sought to identify the cellular dynamics of oligodendrocytes during AD progression. To this end, we searched for a population of disease-associated oligodendrocytes (DAOs) in an App knock-in (KI) AD mouse model during AD progression using droplet-based single-cell RNA sequencing. We identified previously undiscovered Mbp+Cd74+ oligodendrocytes during AD progression in mouse and postmortem AD brains using unbiased single-cell clustering, suggesting that these oligodendrocytes are associated with AD pathogenesis. Disease progression was linked to specific transcriptomic dysregulation patterns associated with Erk signaling in oligodendrocytes. Further, our findings demonstrate that inhibition of Erk signaling in these oligodendrocytes effectively suppresses disease progression and AD phenotypes in App KI AD mouse models. Therefore, elucidating the molecular mechanisms that drive DAO alterations may provide a useful basis to uncover targets for AD treatment.

## Results

### Identification of AD-associated oligodendrocytes in an App KI mouse model

To identify AD-associated cellular components in oligodendrocytes, transcriptome profiling was conducted at the single-cell level via single-cell RNA sequencing using 1-, 3-, and 6-month-old wild-type (WT) and App KI mice (App^NL-G-F^) harboring the Swedish and Beyreuther/Iberian and Arctic mutations in the App gene locus^22^. After clustering (Supplementary Fig. 1a–e), a total of 37,943 cells were annotated into 23 clusters using a predefined set of marker genes^23–25^ (Supplementary Fig. 1c). Afterward, the 6,984 oligodendrocytes were sub-sorted based on the integrated data.

**Fig. 1:**
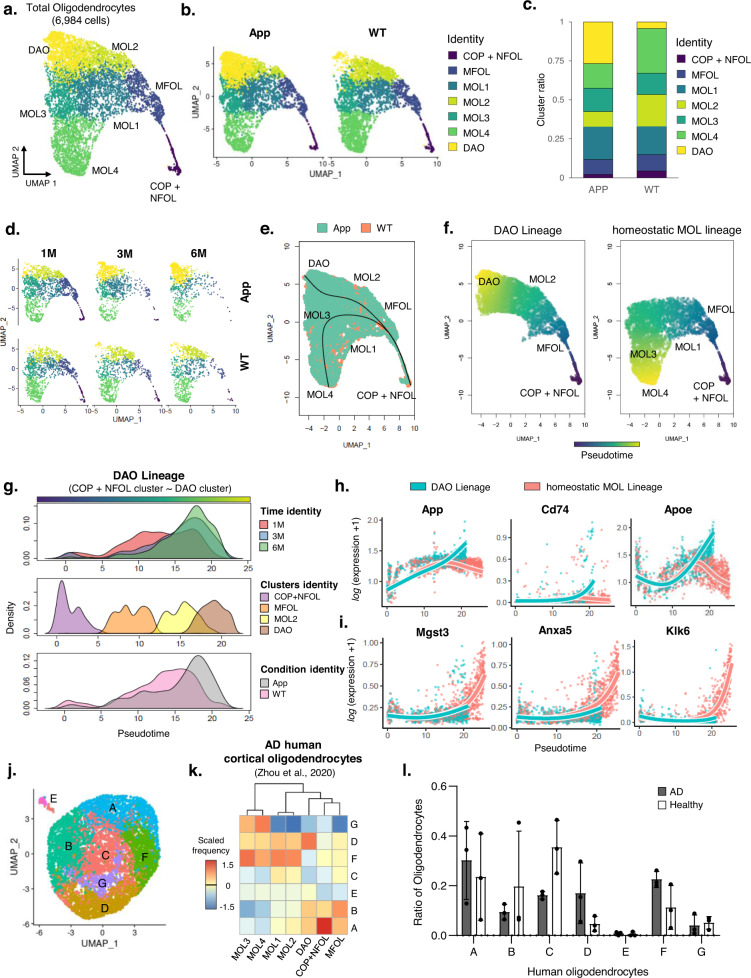
Single-cell RNA-seq reveals disease-associated oligodendrocytes (DAOs) in App KI mice. **a** UMAP plot showing oligodendrocyte clusters in the hippocampal region of WT and App^NL-G-F^ mice. **b** UMAP plot showing oligodendrocyte clusters across conditions. **c**, **d** Distribution of oligodendrocytes from 1-, 3, 6-month-old WT, and App^NL-G-F^ mice. **e** Two lineages of oligodendrocytes with a cluster-based minimum spanning tree with line. **f** Continuous trajectory projection by slingshot-based UMAP plot showing two lineages. **g** Density plot of the DAOs continuous trajectory across three-time points, clusters, and conditions. **h** Visualization of smoothed gene expression patterns of the DAO lineage, plotted on pseudotime. **i** Visualization of smoothed gene expression patterns of the homeostatic mature oligodendrocyte lineage, plotted on pseudotime. **j** UMAP plot showing all human oligodendrocytes merged with the healthy control (*n* = 3) and postmortem AD (*n* = 3). **k** Analysis of expression prevalence between human and mouse with top expression signature markers. The color bar indicates the expression prevalence of human oligodendrocytes A, B, C, D, E, F and G (columns) mapped to the mouse COP + NFOL, MFOL, MOL1, MOL2, MOL3, MOL4, and DAO clusters (rows). **l** Bar plot showing the ratio of the proportion of total oligodendrocytes in healthy control (*n* = 3) and postmortem AD (*n* = 3). Source data are provided as a Source Data file. WT wild-type, AD Alzheimer’s disease, M month.

To reveal heterogeneity within oligodendrocytes, we grouped the aforementioned oligodendrocytes into six classes described in a published database^26^, as follows (Fig. 1a and Supplementary Fig. 2a–d and 3a): (1) Differentiation-committed oligodendrocyte precursors (COPs) and newly formed oligodendrocytes (NFOL) that express Sox6, Pdgfra, and Cspg4. (2) Myelin-forming oligodendrocytes (MFOL) that express Ctps, Ablims, and Thbs3. (3) Mature oligodendrocytes 1, 2 (MOL 1, 2) that express Il33, Ptgds, and Opalin. (4) Mature oligodendrocytes 3, 4 (MOL 3, 4) that express Klk6, S100b, Anxa5, Cd59a and Mgst3. Moreover, we discovered an additional oligodendrocyte cluster, which gradually increased in the 3-, 6-month-old App KI mice (Fig. 1b–d and Supplementary Fig. 2e). This cluster was present in the UMAP plot even with down-sampled data (400, 1,200, and 2,400 cells) (Supplementary Fig. 2f). To identify transcriptional patterns of this cluster, we conducted differential expression gene analysis by using the FindAllMarkers function in the Seurat package (Supplementary Data 1). Remarkably, we found that this cluster shared some signatures with MOL2, while Alzheimer’s disease risk factors such as C4b, Apoe and Cd74^2,27,28^ are strongly expressed (Supplementary Fig. 2c, 3a, 3b, and Source data file). Therefore, we designated this cluster as disease-associated oligodendrocytes (DAOs).

We further performed pseudotemporal ordering of the transcriptional dynamics of oligodendrocyte clusters to capture the molecular dynamics of DAOs in AD progression. Along with a cluster-based minimum spanning tree (MST) analysis, we observed that the MOL2 cluster reflected a transition-like intermediate state between the DAOs and MFOL, suggesting MOL2 primarily contribute to the DAO cluster (Fig. 1e, f). We further confirmed the lineage transitions of DAOs and homeostatic MOLs from COP and NFOL in control and AD brains (Fig. 1e–g and Supplementary Fig. 4), but density from the DAO cluster exclusively appeared in 3 and 6-month-old AD brains (Fig. 1 g). Moreover, we found that the DAO lineage had an increased expression pattern of AD associated gene signatures such as App, Cd74 and Apoe (Fig. 1h), but did not show an increase in MOL signatures such as Mgst3, Anxa5 and Klk6 (Fig. 1i).

We next sought to determine whether DAOs could be identified in the brains of postmortem AD. We reanalyzed previously published single-cell transcriptome datasets from postmortem AD brains (Supplementary Fig. 5a–c)^19^. We identified seven clusters of oligodendrocytes based on distinct transcriptome states in control and postmortem AD oligodendrocytes (Fig. 1j). Moreover, when we compared mouse DAOs with human oligodendrocyte clusters, cluster D of the human oligodendrocytes appeared at a higher frequency with the mouse DAO cluster (Fig. 1k) and had an increased proportion in postmortem AD cortex (Fig. l). Taken together, our results demonstrate the presence of AD-associated oligodendrocytes in the brains of postmortem AD and App KI AD mice.

### Disease-associated oligodendrocytes in App KI mice

**Fig. 2:**
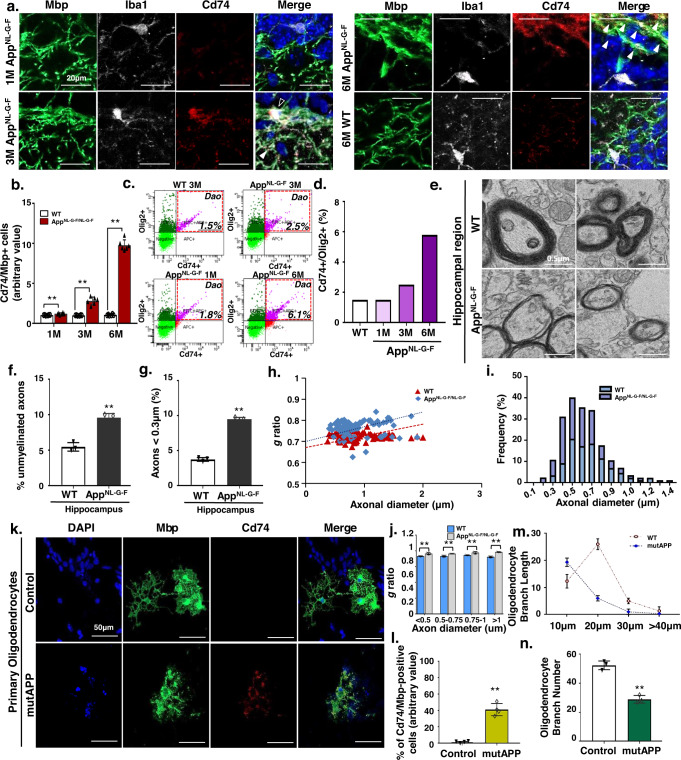
Identification of a novel disease-related oligodendrocyte cluster in Aβ-reactive state. **a** Immunohistochemistry for Mbp (green), Iba1 (white), Cd74 (red), and DAPI (blue) in the DG of WT at 6-months and App^NL-G-F^ mice at 1, 3, and 6-months of age. White, Cd74+oligodendrocyte; Black, Cd74+microglia. **b** Quantification of Cd74/Mbp-positive cells in WT and App^NL-G-F^ mice at 1, 3, and 6-months of age. All data are expressed as mean ± SEM, *n* = 6. **p < 0.01, two-sided Student’s *t*-test. **c** The frequencies of Cd74+ and Olig2+ were determined by FACS analysis. Representative FACS plots are shown. **d** Bar graph illustrating the results of the quantitative FACS analysis of Cd74-positive subsets of the Olig2-positive population. **e** Electron micrographs of axons in the DG from 6-month-old WT and App^NL-G-F^ mice. **f** Percentage of unmyelinated axons in the DG of 6-month-old WT and App^NL-G-F^ mice. All data are expressed as mean ± SEM, *n* = 3. **p < 0.01, two-sided Student’s *t*-test. **g** Percentage of small-caliber axons in DG regions from 6-month-old WT and AD mice. All data are expressed as mean ± SEM, *n* = 3. **p < 0.01, two-sided Student’s *t*-test. **h** Scatter plot of g-ratio values in DG regions from 6-month-old WT and App^NL-G-F^ mice. **i** Distribution of the axon caliber of DG regions from 6-month-old WT and App^NL-G-F^ mice. **j** g-ratio of 6-month-old WT and AD mice according to axon diameter. All data are expressed as mean ± SEM, *n* = 3. **p < 0.01, two-sided Stude*n*t’s *t*-test. **k** Immunohistochemistry for Mbp (green), Cd74 (red), and DAPI (blue) in primary oligodendrocytes with mutAPP treatment. **l** Percentage of Cd74/Mbp-positive cells. Data are expressed as mean ± SEM, *n* = 4. **p < 0.01, two-sided Student’s *t*-test. **m** Branch lengths of mutAPP primary oligodendrocytes by branch length. Data are expressed as mean ± SEM, *n* = 3. *p < 0.05, two-sided Student’s *t*-test. **n** Number of oligodendrocyte branches. Data are expressed as mean ± SEM, *n* = 3. **p < 0.01, two-sided Student’s *t*-test. Source data and *p*-values in **b**, **d**, **f**–**j**, and **l**–**n** are provided as a Source Data file.

Next, we confirmed the presence of DAOs in the hippocampal region of App KI mice. Notably, consistent with scRNA-seq results, we observed a significant increase in Mbp + , Cd74+ oligodendrocytes in 3- and 6-month-old App KI and 6-month-old 5xFAD mice, whereas the number of NeuroD1+ post-mitotic neurons significantly decreased (Fig. 2a, b, Supplementary Fig. 6a–c, and Supplementary Fig. 7a, b). We further confirmed an increase in Cd74+ and Oligo2+ AD-associated oligodendrocytes in App KI mice using flow cytometry (Fig. 2c, d, and Supplementary Fig. 6d).

We further measured axon diameter and myelin thickness to determine the g-ratio. Transmission electron microscopy (TEM) analysis revealed that 6-month-old App KI mice exhibited a thinner myelin layer relative to the axon diameter (Fig. 2e and supplementary fig. 8). Moreover, aberrant myelin and smaller axons (<0.3 μm) were significantly increased in the hippocampus region of 6-month-old AD mice (Fig. 2f, g), suggesting that the g-ratio was substantially elevated in the AD hippocampus compared to control (Fig. 2h). Given that axon diameter is intimately linked to myelin thickness, our results suggest that reduced axon diameter in the hippocampal neurons of AD mice may be due to incomplete myelination (Fig. 2i, j). Additionally, we examined the presence of DAOs upon mutant App overexpression in primary neuronal cultures. After overexpression of mutant APP (mutAPP), we confirmed an increase in Cd74 + Map2+ oligodendrocytes and a reduction in branch length and length in in vitro primary neuronal cultures (Fig. 2k–n). Taken together, our findings demonstrate the presence of Cd74+ DAOs in both in in vitro and in vivo AD conditions.

Additionally, we characterized the gene signature of AD-associated oligodendrocytes. Previous studies showed a significant increase in DAM and DAA populations in aged mice compared to younger adults^10,11^. Consistently, we found a significant increase in Mbp+ and Cd74+ DAOs in the hippocampus of premature progeria (6-month-old) and 13-month-old mice (Supplementary Fig. 9a–d), whereas the number of NeuroD1-positive post-mitotic neurons decreased in the hippocampus of 13-month-old mice (Supplementary Fig. 9e and f). We further confirmed that the genes enriched in DAOs were highly correlated with gene signatures shown in the aged human brain^29^ (Supplementary Fig. 10a, b), indicating that Cd74-positive DAOs increased in the aged brains and had transcriptional profiles more similar to the aged human brain.

### Molecular signature of disease-associated oligodendrocytes

**Fig. 3:**
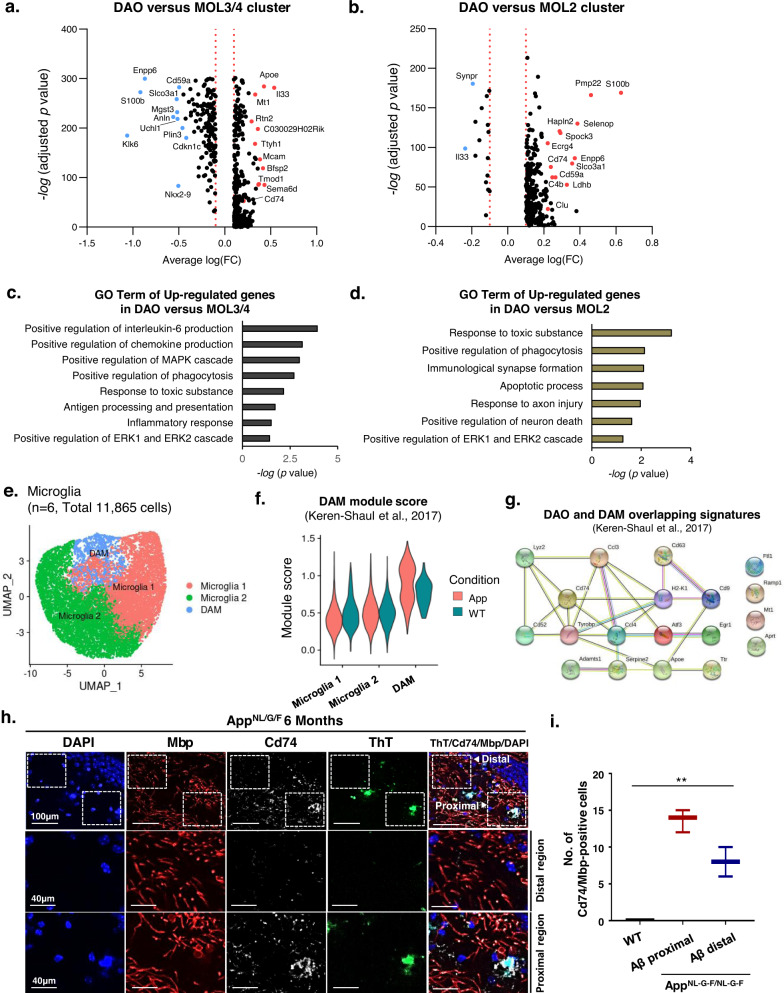
Identification of molecular dynamics in DAOs. **a** Volcano plot showing differentially expressed genes in DAOs compared with MOL3/4 clusters (Wilcoxon rank sum test; two-sided, *log* (fc) threshold > 0.1 and log (fc) threshold < −0.1). **b** Volcano plot showing differentially expressed genes in DAOs compared with the MOL2 cluster (Wilcoxon Rank Sum test; two-sided, *log* (fc) threshold > 0.1 and *log* (fc) threshold < −0.1). **c** Gene ontology of up-regulated genes in DAOs versus MOL3/4 clusters; one-sided *p*-values for Fisher’s Exact test. **d** Gene ontology of up-regulated genes in DAOs versus MOL2 cluster; one-sided *p*-values for Fisher’s Exact test. **e** UMAP *p*lot showing microglia 1, microglia 2, and DAM clusters in age-matched WT and App KI mice. **f** Signature module scores of disease-associated microglia (DAM) enriched in our AD data. Violin plots showing the DAM module score in sub-clusters of WT and App microglia across different conditions (calculated from upregulated DAM genes compared to those of homeostatic microglia)^10^. **g** Gene interaction plot showing 19 overlapping genes between DAM and DAO clusters. Overlapping signatures between DAM^10^ and DAO clusters. **h** Immunohistochemistry for ThT (green), Cd74 (white), Mbp (red), and DAPI (blue) in the hippocampus of 6-month-old App^NL-G-F^ mice. **i** Number of Cd74/Mbp-positive cells from plaque distal and proximal regions. Data are expressed as mean ± SEM (*n* = 3). **p < 0.01, two-way ANOVA with Tukey’s post hoc test. Source data and *p*-values are provided as a Source Data file.

We next explored the unique molecular signatures of DAOs during AD development (Supplementary Data 2). We first examined differentially expressed genes between DAOs and mature oligodendrocytes. In particular, we found significant alternations in the transcriptional profiles between DAOs and MOL 3/4 clusters and GO-term of up-regulated genes in DAO versus MOL3/4 showed the interleukin & chemokine production and ERK signaling (Fig. 3a and c), which was also confirmed during the disease progression in AD mice brain (Supplementary Fig. 11a, b). Next, we examined differentially expressed genes between DAOs and transition-like intermediate state, MOL2 cluster. Consistently, the transcriptional profiles between DAOs and MOL 2 clusters were also dramatically altered and GO-term of up-regulated genes in DAO versus MOL2 identified apoptotic process, axon injury and ERK signaling (Fig. 3b and d). These results suggest that DAOs, unlike MOL 3/4 and MOL2, appeared to have unique transcriptional features such as ERK signaling.

Additionally, we determined the transcriptional correlation between DAOs and DAMs. We first identified microglia with a high DAM module score based on previously published DAM signatures^10^ (Supplementary Fig. 1e, 12a, b), that gradually increased during AD progression (Fig. 3e, f, and Supplementary Fig. 12c). Notably, we found 19 shared genes between the 190 DAO signature genes and 201 signature genes previously described for DAM^10^ (Fig. 3g, hypogeometric *p* < 9.575e-12). A number of these genes - such as Ccl3, Ccl4, Apoe, and Cd74 - are implicated in AD development and aging^30,31^. Moreover, since previous studies showed that DAMs and DAAs were localized around Aβ plaques^10,11^, we examined whether DAOs were also localized in the proximity of Aβ plaques^10,11^. Interestingly, we found that DAOs were mostly located adjacent to Aβ plaques in the AD mouse hippocampus (Fig. 3h, i), suggesting that DAOs (like DAMs and DAAs) are relevant to AD progression.

### Erk1/2 signaling mediates activation of disease-associated oligodendrocytes

**Fig. 4:**
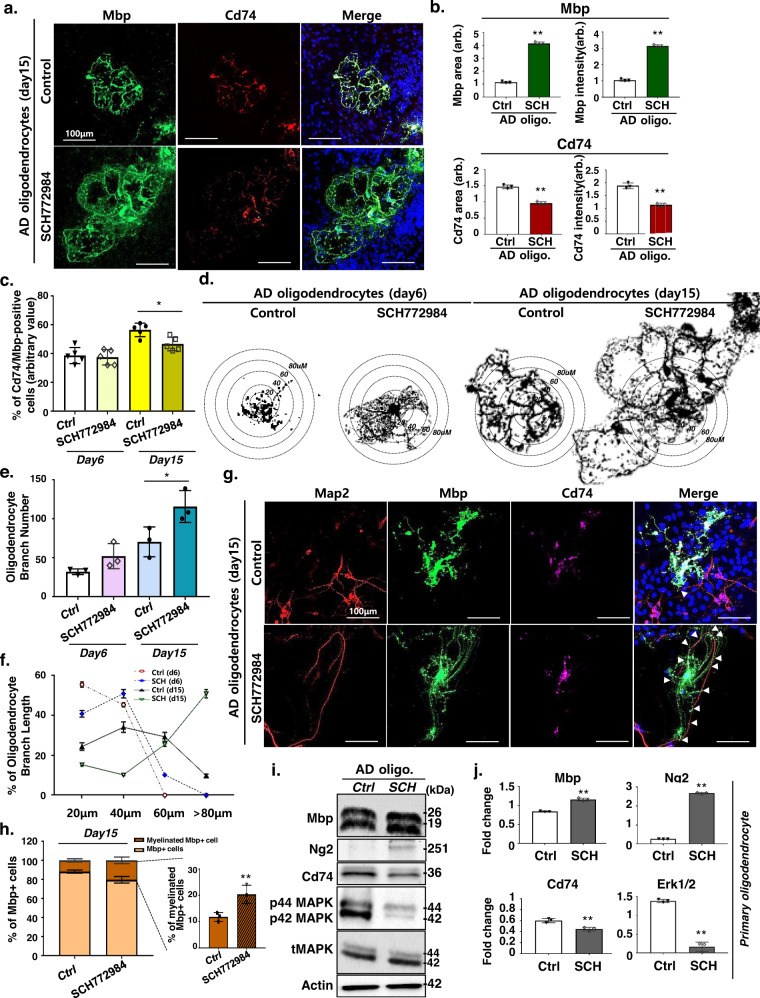
ERK inhibitor rescues AD-associated abnormal oligodendrocytes. **a** Immunohistochemistry of Mbp (green), Cd74 (red), and DAPI (blue) in App^NL-G-F^ primary oligodendrocytes treated with SCH772984 on day 15. **b** Quantification of the intensity of Mbp and Cd74 immunohistochemistry in SCH772984-treated App^NL-G-F^ primary oligodendrocytes. Data are expressed as mean ± SEM, *n* = 3. **p < 0.01, two-sided Student’s *t*-test. *p*-values are provided as a Source Data file. **c** Percentage of Cd74/Mbp-positive oligodendrocytes in control and SCH772984-treated oligodendrocytes on days 6 and 15. Data are expressed as mean ± SEM, *n* = 5, one-way ANOVA coupled with Tukey’s post hoc test (*p* < 0.05). **d** Mature Mbp-positive oligodendrocytes extending branched processes in SCH772984-treated App^NL-G-F^ primary oligodendrocytes at days 6 and 15. Branching was quantified by measuring the size of the branches in the circles. **e** Number of branches in the SCH772984-treated App^NL-G-F^ primary oligodendrocytes. Data are expressed as mean ± SEM, *n* = 3, one-way ANOVA coupled with Tukey’s post hoc test (*p* < 0.05). **f** Branch length distribution in SCH772984-treated App^NL-G-F^ primary oligodendrocytes. Data are expressed as mean ± SEM, *n* = 3. two-sided Student’s *t*-test. **g** Immunohistochemistry for Map2 (red), Mbp (green), Cd74 (purple), and DAPI (blue) in primary neuron/oligodendrocyte co-cultures at day 15. **h** Percentage of myelinating Mbp-positive oligodendrocytes. Data are expressed as mean ± SEM, *n* = 3. **p < 0.01, two-sided Stude*n*t’s *t*-test (*p* = 0.018). **i** Western blot analysis of Mb*p*, Ng2, Cd74, p44/p42 MAPK, tMAPK, and Actin in SCH772984-treated App^NL-G-F^ primary oligodendrocytes. Western blot performed three biological replicates. **j** Quantification of western blot results shown in **i**. Data are expressed as mean ± SEM, *n* = 3. **p < 0.01, two-sided Student’s *t*-test. Source data in **b**, **c**, **e**, **f**, **h**, **i**, and **j** are provided as a Source Data file. *p*-values in **b**, **c**, **e**, **f**, and **j** are provided as a Source Data. arb arbitrary units.

Given that we identified upregulation of MAPK/ERK genes in the DAOs, we next examined the role of MAPK/ERK signaling in AD DAOs. We initially isolated primary oligodendrocyte progenitors from App KI AD mice and cultured them in the absence of neurons in conditions that promoted further differentiation. Supplementary Fig. 13a–c shows efficient inhibition of phosphor 44/42 MAPK signaling in primary oligodendrocytes upon treatment with 200 ng/ml SCH772984, a specific Erk1 and Erk2 inhibitor. Remarkably, oligodendrocytes treated with SCH772984 exhibited a significant increase in Mbp+ branching compared with the controls, whereas the number of Cd74+ cells significantly decreased (Supplementary Fig. 13d, 13e, and Fig. 4a–c). Consistent with these observations, we further confirmed a reduction in Cd74+ and Mbp+ DAOs and an increase in branching upon SCH772984 treatment at 6 and 15 days of differentiation (Fig. 4d–f). We next assessed whether inhibition of ERK/MAPK in DAOs can contribute to myelin formation. After 15 days, the primary neuronal cultures derived from App KI embryonic mice mostly failed to unsheathe the neurons. However, the primary neuronal culture treated with SCH772984 exhibited myelin-like sheaths surrounding the axons, as demonstrated by confocal analysis of Mbp and Map2 expression (Fig. 4g and h). Moreover, upregulation of Mbp and Ng2, as well as a significant reduction of Cd74 and Erk1/2, was confirmed in SCH772984-treated primary neuronal cultures from control and AD KI mice (Fig. 4i and j). These data suggest that ERK/MAPK signaling is a key driver of DAO production and differentiation in the AD environment.

Additionally, we examined potential interactions between DAOs and other neuronal cell types and also whether other cell types can contribute to the AD pathology, especially in the light of dysregulated Erk1/2 signaling. First, in order to examine the potential interactions between DAOs and other neuronal cell types, AD DAOs were co-cultured with AD neurons, astrocytes, and microglia treated with SCH772984 (Supplementary Fig. 14a). We found that AD DAOs with other neuronal cell types did not increase the number of Mbp+ branches in the presence and absence of SCH772984 treatment (Supplementary Fig. 14b–f), suggesting that inhibition of ERK/MAPK signaling in other neuronal cell types did not contribute to the phenotypic changes in DAOs (Fig. 4d–f). Moreover, to investigate whether other neural cell types can contribute to the AD-associated phenotypes via ERK signaling, each of the neurons, microglia, astrocytes and oligodendrocytes isolated from AD mice were subjected to SCH772984 treatment, and AD pathology was determined after co-culture with the remaining untreated AD neuronal cell types (Supplementary Fig. 15a). Remarkably, we found that ERK1/2-inhibited oligodendrocytes significantly diminished the Aβ42/40 ratio in the AD primary neuronal cultures (Supplementary Fig. 15b). Also, we observed a decrease in the amyloidogenic processing marker C99 in the AD neuronal cultures with SCH772984 treatment (Supplementary Fig. 15c–e), indicating that AD oligodendrocytes predominantly contribute to the pathophysiology of AD via ERK signaling.

### Inhibition of Erk1/2 in disease-associated oligodendrocytes suppresses AD phenotypes in the mouse brain

**Fig. 5:**
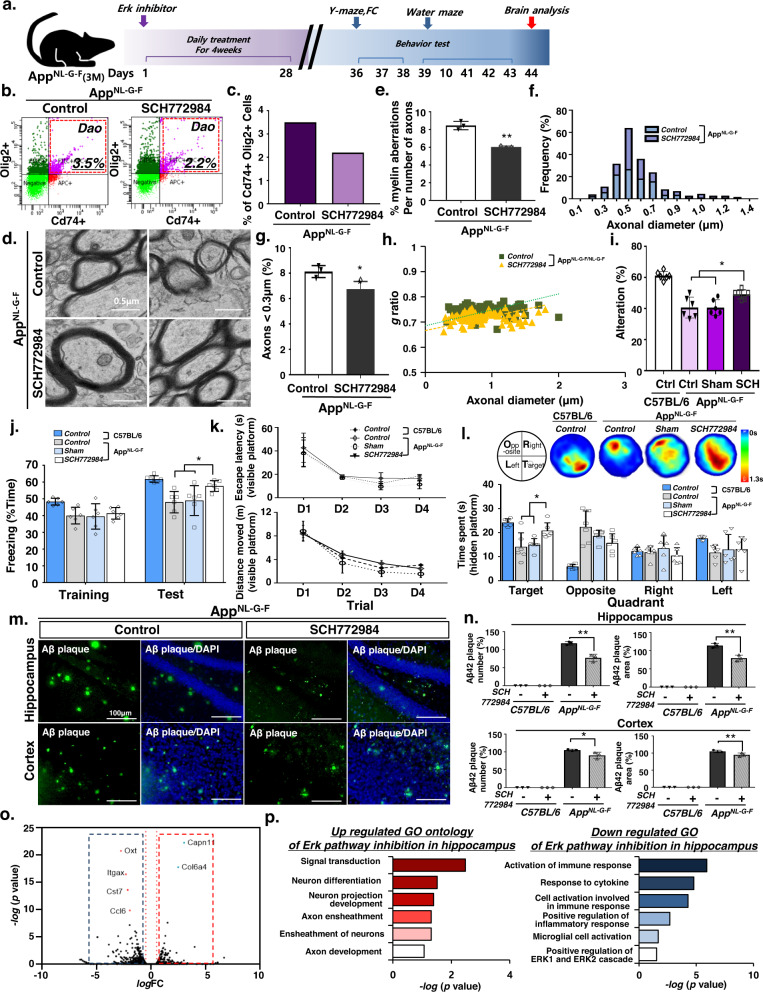
ERK1/2 inhibition rescues cognitive decline in AD mice via DAO regulation. **a** Schematic diagram showing the ERK inhibitor treatment approach. Mice were treated with ERK inhibitors via a daily intraperitoneal injection for 4 weeks. **b** The frequencies of Cd74+ and Olig2+ were determined by FACS analysis. Representative FACS plots are shown. **c** Bar graph showing quantitative FACS analysis of Cd74-positive subsets of the Olig2-positive population. **d** Electron micrographs of axons in the DG from App^NL-G-F^ mice. **e** Percentage of aberrant myelinated axons. Data are expressed as mean ± SEM, *n* = 3. **p < 0.01, two-sided Student’s *t*-test. **f** Distribution of axon caliber. **g** Percentage of small-caliber axons. Data are expressed as mean ± SEM, *n* = 3. *p < 0.05, two-sided Student’s *t*-test. **h** Scatter plot of g-ratio. **i** Y-maze test of App^NL-G-F^ mice. Data are expressed as mean ± SEM (*n* = 6). *p < 0.05, one-way ANOVA with Tukey’s post hoc test. **j** Fear conditioning test of mice. Data are expressed as mean ± SEM (*n* = 6). *p < 0.05, two-way ANOVA with Tukey’s post hoc test. **k**, **l** Water maze test of App^NL-G-F^ mice. **k** Escape latency and distance moved during trial. Data are expressed as mean ± SEM (*n* = 6). one-way ANOVA with Tukey’s post hoc test. **l** The upper panel illustrates visited area, and the bottom panel presents quadrant occupancy quantification analysis. Data are expressed as mean ± SEM (*n* = 6). *p < 0.05, two-way ANOVA with Tukey’s post hoc test. **m** Immunohistochemistry for ThT in App^NL-G-F^ mice. **n** Percentage of Aβ plaque number and area in Fig. 5m. Data are expressed as mean ± SEM. *p < 0.05 and **p < 0.01, one-way ANOVA with Tukey’s post hoc test. **o** Volcano plot of genes based on RNA-seq analysis. The threshold for determining differentially up- and down-regulated gene expression is indicated by blue (downregulated) and red (upregulated) boxes (one-sided, *log* (*p*-value) > 5, *log* (FoldChange) > 1.5). **p** Gene ontology enrichment analysis of significantly up- or down-regulated genes; one-sided *p*-values for Fisher’s Exact test. Source data and *p*-values in **e**, **g**, **i**–**l**, and **n** are provided as a Source Data file.

Given the increase in Map+Cd74+ DAOs in the AD mouse brain, we next tested whether inhibition of Erk1/2 signaling attenuates DAO activation, which may alleviate memory impairments in AD mouse models. SCH772984 was administered to 3-month-old App KI AD mice via intraperitoneal injection once daily for 4 weeks, after which behavioral and biochemical analyses were conducted 5 weeks later (Fig. 5a). Consistent with previous results, systemic administration of SCH772984 significantly reduced the occurrence of DAOs in the hippocampus of App KI mice (Fig. 5b, c, and Supplementary Fig. 16a–d) but not Cd74 + Iba1+ microglia (Supplementary Fig. 16c, d). We observed a significant decrease in the number of oligodendrocytes with aberrant myelin, and increased axon diameter in the SCH772984-treated AD mice (Fig. 5d–f). SCH772984 injection decreased the occurrence of small axons (<0.3 μm) in AD mouse brains (Fig. 5g). Moreover, the SCH772984 treatment decreased the g-ratio (i.e., the ratio of the inner axonal diameter to the total outer diameter) in the AD mouse brain (Fig. 5h). Therefore, our results suggest that SCH772984 treatment inhibited Erk1/2 signaling in AD mice, thus restoring normal myelin formation by inhibiting the occurrence of abnormal DAOs.

We next conducted cognitive behavior tests in SCH72983-treated AD mice to verify the therapeutic value of this compound. First, SCH72983-treated AD mice were subjected to the Y-maze test to assess spatial working and reference memory. Remarkably, SCH772984-treated AD mice exhibited an enhanced alternation performance behavior compared to the AD control mice (Fig. 5i). Moreover, we confirmed that AD mice treated with SCH772984 spent significantly more time exhibiting freezing behaviors during the contextual fear conditioning test (Fig. 5j). We additionally tested the performance of SCH772984-treated AD mice in the Morris water maze test. On the test day without a platform, SCH772984-treated AD mice spent more time in the target quadrant compared to the control AD mice (Fig. 5k, l), suggesting that targeting DAOs with SCH772984 might be a promising therapeutic strategy to rescue AD-associated cognitive behaviors in AD mice. Moreover, we confirmed a decrease in the number of Aβ plaques (Fig. 5m, n), coupled with an increase in the number of NeuroD1+ cells in the SCH772984-treated AD mouse hippocampus (Supplementary Fig. 16e, f).

To confirm the suppression of Erk signaling in the SCH772984 treated AD hippocampus, differential gene expression analysis in the hippocampal region in AD mice was determined after SCH772984 treatment. We found that the expression levels of 39 genes were significantly upregulated, whereas the expression levels of 114 genes were significantly downregulated (Fig. 5o). GO term enrichment analysis indicated that the most enriched upregulated gene signatures were associated with neuronal development, whereas downregulated genes were strongly associated with Erk1/2 signaling regulation (Fig. 5p), suggesting that inhibition of Erk signaling with SCH772984 may be a promising strategy to treat AD.

## Discussion

Oligodendrocytes are glial cells that form myelin in the CNS, and similar to other glial cells, maintain and support nervous system cell function^32,33^. Oligodendrocytes wrap myelin around axons to form the myelin sheath, which prevents ion leakage and maintains axon potential^34^. Several studies have reported that myelin degradation occurs with aging and accelerates over time^35^. Additionally, oligodendrocyte dysfunction may be linked to the pathogenesis of neurodegenerative diseases such as AD^18,36^ or Parkinson’s disease (PD)^37^. The AD mouse model has been reported to exhibit significant changes in myelination patterns and oligodendrocyte status prior to the occurrence of Aβ pathology. Moreover, myelin degradation promotes the accumulation of beta-amyloid fibrils, which in turn adversely affects neuron maintenance and support^14,38–40^. Moreover, myelin degradation stimulates the progression of Aβ pathology, thereby further disrupting myelin^41^. For example, inhibition of the differentiation of oligodendrocyte precursor cells in the mouse brain resulted in impaired learning^42,43^. Moreover, an increase in the occurrence of immature or abnormal oligodendrocytes coupled with a decrease in oligodendrocytes^14–16^ was observed in AD mouse models such as PS1 KI^15^, 3xTG-AD^14^, and APPPS1^16^. Furthermore, several studies have reported that Aβ is toxic to oligodendrocyte precursor cells and/or oligodendrocytes^17,18^. Therefore, these studies suggest that dysfunction of oligodendrocytes or their precursor cells during the process of myelin production and remyelination are closely associated with AD onset and progression.

Our study reports the existence of previously uncharacterized AD-associated oligodendrocyte subtypes, elucidated via single-cell expression profiling analysis. Understanding the molecular and cellular changes of DAOs might provide a basis for the development of therapeutic approaches to treat AD. Aberrant gene signatures in oligodendrocyte and oligodendrocyte precursor cells in AD have been previously reported. These studies provided a critical starting point for the identification of DAO subtypes. Previous studies have relied on single-cell analysis of the AD brain at a single time point. In contrast, our single-cell analysis approach was conducted at different time points during AD progression, thus allowing for the identification of DAOs. Two main immune cell types, DAM and DAA, were previously reported to be involved in AD pathobiology, as demonstrated by single-cell analysis^10,11^. DAM and DAA have been identified in both AD mice and humans and their occurrence increased markedly as the disease progresses. These studies revealed the transcriptional signatures of DAM and DAA during AD progression. Our results also elucidated similar transcriptional signatures in DAOs. Therefore, the systematic identification of molecular networks among DAM, DAAs, and DAOs may allow for the identification of targets for AD treatment.

It has been suggested previously that the role of the MAPK/ERK pathway in AD is related to the inflammatory response, Aβ deposits, and mitochondrial dysfunction. These results suggest that the MAPK/ERK pathway plays an essential role in the noxious events that lead to AD induction. Together, these findings expand upon previous studies of oligodendrocytes, suggesting a pathogenic role of demyelination, in addition to the essential function of the MAPK/ERK pathway. However, the specific mechanisms by which MAPK/ERK mediates oligodendrocyte impairment remain unclear.

In summary, the findings of our single-nucleus transcriptome analysis constitute a useful resource for understanding the pathological roles of oligodendrocytes in AD. Moreover, our results indicated that the regulation of the MAPK/ERK pathway could restore saltatory neuron conduction by inducing remyelination. Therefore, our study establishes a theoretical basis for the future identification and characterization of therapeutic targets for the treatment of neurodegenerative diseases.

## Methods

### Ethics statement

All experimental procedures and care of animals in this study were carried out according to the Institutional Animal Care and Use Committee at Dongguk University.

### Animal experiments

All animal experiments were approved by the Institutional Animal Care and Use Committee at Dongguk University and performed in accordance with institutional guidelines (IACUC-2021-042-2). App KI (*App*^*NL-G-F/NL-G-F*^; Swedish (NL), Beyreuther/Iberian (F), and Arctic (G) mutation knock-in) transgenic mice were obtained from the RIKEN Brain Science Institute^22^. And B6C57 mice were obtained from Daehan Biolink Co. Ltd.. All mice were maintained in a controlled condition of 12 h light (8 a.m.–8 p.m.)/12 h dark (8 p.m.–8 a.m.) cycle. And all mice were kept in temperature at 22–23 °C, humidity 50-60%, and air management systems which control air flow. Mice lines were checked daily. Behavior assays were conducted using 4-month-old App KI mice (*n* = 6 male mice per group) and B6C57 mice (*n* = 6 male mice per group). Concretely, we conducted the Y-maze, fear conditioning, and water maze assays. The Y-maze assay was performed to characterize the short-term memory of AD mice. Alternation was tested using a Y-shaped three-open arm maze (30 × 5 cm). Mouse behaviors were recorded for 10 min. The fear conditioning assay was conducted for 2 days. On the day of the trial, the mice were placed in a fear-conditioning chamber and submitted to a mild electric shock (1 s, 0.7 mA). After 24 hrs of the initial test, freezing behaviors were evaluated to assess fear conditioning. Water maze tests were performed to evaluate learning and memory. Water maze tests were conducted using a circular water maze and recorded for 1 min. For the visible platform trial, the mice were trained for 4 days with three trials per day. During the unviable platform test, the time spent in each quadrant was recorded for 1-min periods. All App KI mice were randomly selected for the behavior experiments. Behavior and image analyses were blinded and performed independently. Histological analyses were also blinded and performed independently.

### Single-cell isolation and scRNA-seq

We followed the previous reports for the successful isolation of oligodendrocytes for single-cell RNA seq (Marques and Zeisel et al. 2016), which showed the defined OL subtypes (OPC, NFOL, MFOL, and MOL). We also confirmed several clusters of oligodendrocyte subtypes by using this single-cell RNA seq method. Briefly, brain samples from male wild-type and App-KI transgenic mice were prepared in the same single-cell isolation batch to minimize potential batch effects. Hippocampus tissues from 1-, 3-, and 6-month-old wild-type and App-KI mice were then dissected (Supplementary Data 3). One mouse per age and genotype were used for library preparation. Experiments and randomly assigned to each group. The dissected tissues were then minced into ~1 mm^3^ pieces using sterile razor blades and detached by incubation for 20 min in a solution containing 0.01% papain (LS003126, Worthington Biochemicals) and 0.01% DNase I (9003-98-9, Worthington Biochemicals). The cell suspension was combined with an equal volume of 0.5% BSA + DPBS and filtered through a 70 µm strainer. The filtered cells were counted using a hematocytometer and diluted to a 1000 cells/µL concentration before carrying out single-cell capture on a 10X Genomics Single-Cell 3' system. The 10X library construction protocol was followed without alteration. Single-cell cDNA libraries from individual cells were pooled and sequenced 5 million reads per sample on a HiSeq 4000 sequencer.

### ERK1/2 inhibitor treatment

The ERK1/2 inhibitor (SCH772984, HY-50846) from MedChemExpress was dissolved in DMSO. Cells were treated with a dose of 200 nM ERK1/2 in vitro, whereas a 25 mg/kg ERK1/2 concentration of inhibitor was injected into the 3-month-old App KI mice (*n* = 12 male mice per group) once daily for 4 weeks for the in vivo treatments.

### Cell culture

For primary oligodendrocyte culture, the primary oligodendrocytes were derived from 2-day ICR fetuses (DBL Co., Ltd., Eumseong-gun, Chungcheongbuk-do, South Korea) or App KI mice. Mouse primary oligodendrocyte progenitor cells (OPC) were prepared from the cortex of 2-day mouse fetuses, as previously described^44,45^. Purified OPCs were differentiated to oligodendrocytes in DMDM/F12 (11330032, Gibco) supplemented with B27 (A3653401, Gibco), L-glutamine (25030149, Gibco), penicillin-streptomycin (15070063, Gibco), insulin (I6634, Sigma), FBS (F0900-050, GenDEPOT), transferrin (T2036, Sigma), and OL supplement (DMEM, BSA, progesterone, putrescine, sodium selenite, 3,3,5-Triiodo-L-thyronine). Oligodendrocyte/neuron co-cultures were performed as previously described^46,47^. Oligodendrocytes were seeded onto day 2 cultured neurons and cultured with primary neuron and oligodendrocyte culture medium at a 1:1 ratio. The experimenter was not blinded to treatment. None of the experiments were excluded from our data.

### Magnetic activated cell sorting (MACS)

P1 mice were sterilized with 70% ethanol and rinsed three times in PBS (D8662, Sigma). Mouse primary neural cells were prepared from the cortex and hippocampus of the brain. Neural cells were dissociated into single-cell suspension with gentleMACS Dissociator (Miltenyi Biotec) according to the manufacturer’s protocol. Briefly, prepared brain samples were added with enzyme mix and incubated followed by the gentleMACS dissociator program. Incubated samples were applied to the cell strainer and washed with HBSS. Single-cell suspended samples were purified with MACS and MACS Anti-O4 MicroBead (130-094-543, Miltenyi Biotec), Anti-ACSA-2 MicroBead (130-097-678, Miltenyi Biotec), Anti-CD171 (L1CAM) MicroBead (130-101-549, Miltenyi Biotec), and Anti-CD11b MicroBead (130-049-601, Miltenyi Biotec) according to the manufacturer’s protocol.

### Cell viability

MTT assays were conducted to determine cytotoxicity. 5×10^4^ cells were seeded on a 24-well plate. After 24 hrs, the cells were incubated with varying concentrations of SCH772984. The cells were then supplemented with 5 mg/ml MTT (thiazolyl blue tetrazolium bromide, M2128, Sigma) and incubated for 4 hrs at 37 °C. DMSO (D8418, Sigma) was then added to the cells after removing the MTT solution. Absorbance was measured at 570 nm using a microplate reader (BioRad, Hercules, CA, USA).

### Immunohistochemistry

Immunohistochemistry was performed on cells and sections of App KI mice and B6C57 mice brains. Mice were perfused with PBS followed by 4% paraformaldehyde. Brains were post-fixed with 4% paraformaldehyde overnight followed by 30% sucrose for 48 hrs. Fixed mouse brains were sectioned into 35 μm slices using a Leica vibratome (Leica VT 1000 S, Nussloch, Germany). The sectioned brains and cells were blocked for 20 min and incubated with appropriate primary anti-Cd74 antibodies (1:250, PA5-22113, Invitrogen; 1:250, SC-6262, Santacruz), anti-Mbp antibodies (1:250, ab7349, Abcam), anti-Map2 antibodies (1:250, 4542 s, Cell signaling), and anti-NeuroD1 antibodies (1:250, PA5-47381, Invitrogen). The cells or sectioned brains were then washed three times (5 min per wash) with PBS. Next, the cells and brains were incubated with secondary antibodies for 2 h at room temperature (donkey anti-Rat IgG secondary antibody, Alexa Fluor™ 488 [A-21208, Invitrogen]; goat anti-rat IgG secondary antibody, Alexa Fluor® 594 [ab150160, Abcam]; goat anti-rabbit IgG secondary antibody, Alexa Fluor™ 647 [A-21244, Invitrogen]; goat anti-rabbit IgG secondary antibody, Alexa Fluor™ 594 [A-11012, Invitrogen]; goat anti-rat IgG secondary antibody, Alexa Fluor™ 594 [A-11007, Invitrogen]; goat anti-mouse IgG secondary antibody, Alexa Fluor™ 594 [A-11005, Invitrogen]). Afterward, the samples were once again washed three times (5 min per wash) with PBS. Then, the samples were counterstained with 1 μg/mL 4',6-diamidino-2-phenylindole (DAPI, 1:1000; Invitrogen, Carlsbad, CA, USA) for 5 min at room temperature. We counted the Mbp+ and Cd74+ area and intensities on the same number of Mbp+ cells and Cd74+ cells per well. We quantified the area or intensity for each group in three wells. For thioflavin T (ThT) staining, 10 brain samples per brain were stained with ThT in 50% ethanol for 7 min. After washing the samples with PBS, samples were counter-stained with DAPI. To quantify plaque depositions, brain levels were analyzed in the hippocampal dentate gyrus (DG) (*X* = −2.06, *Y* = ± 1, *Z* = −2.125) and cortical (*X* = −2.06, *Y* = ± 2, *Z* = −0.75) regions of ‘Mouse Brain Atlas’ by George Paxinos in three animals per group. Fixed thickness 35 um tissue sections were maintained at regular intervals. The stained samples were imaged using a Zeiss LSM 700 confocal microscope (Carl Zeiss, Thornwood, NY, USA) with excitation using 488, 594, and 647 nm lasers and the same exposure time. Images were automatically aligned using the stitching tool in ZEN software (Zeiss). Immunohistochemistry was quantified with ImageJ (NIH) software. We quantified the average number or intensity of all samples for each group with three or more wells or slices. The experimenter was not blinded to treatment. None of the cell cultures were excluded from our analyses.

### Western blot analysis

For western blot analysis, the samples were mixed with RIPA buffer (1% NP-40, 0.5% DOC, 0.1% SDS, and 150 mmol/L NaCl in 50 mmol/L Tris, pH 8.0; R0278, Sigma-Aldrich) and 1x proteinase inhibitor and homogenized. Next, the samples were mixed with 5x loading buffer and boiled at 100 °C for 10 min. Ten micrograms of extracted proteins were used for the western blot analysis. The extracted proteins were separated via 12% sodium dodecyl sulfate-polyacrylamide gel electrophoresis (SDS-PAGE) and blotted onto nitrocellulose membrane (10600001, GE Healthcare). The membrane was then incubated with appropriate primary anti-p44/42 MAPK antibodies (1:1000, #4695, Cell signaling), anti-Mbp antibodies (1:1000, ab7349, Abcam), anti-Ng2 antibodies (1:1000, MA5-24247, Invitrogen), anti-APP C-terminal antibodies (A8717, Sigma), anti-Cd74 antibodies (1:1000, PA5-22113, Invitrogen; 1:1000, SC-6262, Santacruz), and anti-beta-actin antibodies (1:1000, LF-PA0207, Abfrontier) overnight at 4 °C. After cells were incubated with the primary antibodies, they were washed three times with PBS. Then, samples were incubated with each suitable secondary for 2 h at room temperature (goat anti-rabbit IgG-HRP [LF-SA8002, AbFrontier]; goat anti-mouse IgG HRP [LF-SA8001, AbFrontier]; goat anti-rat IgG-HRP [LF-SA8003H, AbFrontier]). The resulting bands were visualized with an ECL kit (DG-WF200; Dogen). All experiments were performed at least three times. Each band was quantified with ImageJ (NIH) software. The experimenter was not blinded to the treatment. None of the experiments were excluded from our analyses.

### Transmission electron microscopy (TEM) analysis

Morphological analysis was conducted using transmission electron microscopy (TEM). App KI mice and B6C57 mice brain tissues were fixed in 2.5% glutaraldehyde in 0.1 M phosphate buffer (pH 7.3) at 4 °C for 24 h. The embedded tissue was sliced at a 5 μm thickness. Sectioned samples were images were acquired using an FEI-Tecnai G2 Spirit bio transmission electron microscope at the Korea Basic Science Institute (Daejeon, Korea). For counting myelin aberration in axons, we counted (1) abnormal myelin outfoldings, (2) balooned myelin, (3) degenerated sheaths, and (4) excess cytoplasm in the inner loop. And 200 axons were counted in each of brain region for our analysis

### Fluorescence cytometry

3- and 13-month-old B6C57 and 1-, 3-, and 6-month-old App-KI transgenic mice were sacrificed in a CO_2_ chamber and their brains were immediately put on ice. Pooled hippocampus tissue was digested with papain and DNase I according to the Papain Dissociation System Kit instructions (LK003150, Worthington) to maximize cell viability. The tissues were then treated with a combination of anti-Olig2 (Goat, R&D Systems, 1:500) and anti-Cd74 (Rabbit, Invitrogen, 1:1000) antibodies. Stained cell suspensions were sorted using a BD FACSAria software in a BD FACSAria^TM^ III using a 120 µm nozzle at 20 psi.

### Bioinformatic analysis

Alignment and quality control of transcriptomic data: The 10X Genomics CellRanger v3.0.2 software was used to analyze all datasets. Fastq files were mapped and aligned to the mouse genome (mm10) with the STAR software using all default options. And we removed cells, which have mitochondrial gene content of >10% and filtered cells that have unique feature counts over 6000 or <200.

Dimensionality reduction and clustering of scRNA-seq data: To integrate the datasets to find cell-type-specific responses from App knock-in, we conducted an integrated analysis using the IntegrateData function of the Seurat package (v3.2.2). Using ‘RunUMAP,’ Seurat’s non-linear dimensional reduction algorithm, we clustered cells using variable genes. We then defined each cluster from a public hippocampus database and subsetted oligodendrocyte clusters from entire clusters using the SubsetData function of the Seurat package. Subsetting of oligodendrocytes: some genes suffered from a high occurrence of dropout events and thus exhibited a low signal-to-noise ratio. To solve this, before integrating oligodendrocytes data of WT and App KI, the SAVER software (version 1.1.1) was used to obtain imputed expression profiles of oligodendrocyte clusters. Multidimensional scaling (MDS) of oligodendrocytes was performed using the cmdscale function of the Seurat package. Afterward, Slingshot was used to computationally delineate the AD progression of oligodendrocytes and order them in pseudotime^48^. The oligodendrocyte dataset of Seurat was then converted into SingleCellExperiment objects. Slingshot trajectory analysis was conducted using the Seurat clustering information with dimensionality reduction obtained by MDS.

DAO molecular dynamics during AD progression: Slingshot was used to conduct trajectory analysis^48^. Briefly, dimensional reduction was conducted using the MDS of the oligodendrocyte subsets, after which PCA was performed for individual or integrated datasets. The Seurat objects were then transformed into SingleCellExperiment objects. Slingshot trajectory analysis was performed using the Seurat clustering information with dimensionality reduction produced by MDS.

Differential expression analysis: differentially expressed signatures were calculated using the likelihood-ratio test in the FindMarkers function (ident.1 and ident.2) and visualized using volcano plots. DAM and DAO signatures: the list of DAM signatures was taken from the literature^10^ and the DAM signature was defined as all genes with −log(*p*-value) > 10, resulting in 201 genes. Additionally, our DAM signatures derived from this database were obtained by comparing the DAM cluster with homeostatic microglia using the FindMarkers function in Seurat. The AddModuleScore function in the Seurat package module was used to score the microglia clusters by subtracting the expression of published DAM signatures. To obtain the DAM signature from our dataset, we compared expression values between high module score microglia and normal microglia using the FindMarkers function (min.pct = 0.1 and *log*fc.threshold = 0.1, *p*-value < 0.05). The list of DAO signatures was obtained by comparing the expression between the DAO cluster and the rest of the clusters (min.pct = 0.25, *log*fc.threshold = 0.1, *p*-value < 0.05). Gene-gene functional links within the shared DAM-DAO signature genes were visualized using the STRING database, setting edges to reflect the confidence of the prediction.

Transcriptome analysis of oligodendrocytes between AD mouse and postmortem AD brains: scRNA-seq raw data from dorsolateral PFC (dorsolateral prefrontal cortex) of healthy control and postmortem AD (male/86–93 years old) were obtained using the published cluster annotations and other publications to select oligodendrocytes in the dorsolateral PFC^19^. Briefly, postmortem brain tissues from 10 postmortem AD brains with TREM2 R62H variant, 11 postmortem AD brains with TREM2 common variant, and 11 age-matched controls from the Rush cohort were analyzed by scRNA-seq. Sex was balanced between AD and control individuals, with four males and seven females in AD(CV) and control group, and five males and five female in TREM2 R62H^19^. To compare the human and mouse oligodendrocytes, mouse genes were first mapped to human genes using annotations from a complete list of human and mouse homologs from the Mouse Genome Database (MGI). The human data were re-analyzed following the steps described for the mouse data, including scaling, normalization, variable gene detection, clustering, and visualization using Seurat v.3.2.2. Finally, cluster identity was assigned to the human clusters using the CCA approach (Seurat v.3.2.2). To compare human and mouse expression prevalence, top expression marker genes (18~20 genes) were selected from the mouse dataset. For the selection of top expression signature markers, the Seurat FindMarkers function was coupled with the “bimod” likelihood-ratio test.

### Statistical analysis

All data were presented as the mean ± SEM of three or more independent experiments. Statistical analysis was carried out using the SPSS 18.0 software (SPSS Inc., Chicago, Ill., USA). *p*-values <0.05 were considered statistically significant (*p < 0.05 and **p < 0.01). No statistical methods were used to pre-determine sample sizes; however, our sample sizes were similar to those reported in previous publications^49–56^. Data collection and analysis were randomized and blinded.

### Reporting summary

Further information on research design is available in the Nature Portfolio Reporting Summary linked to this article.

## Supplementary information

Supplementary information

Description of Additional Supplementary Files

Supplementary data 1

Supplementary data 2

Supplementary data 3

Reporting Summary

## Data Availability

The mouse single-cell RNA-seq datasets generated in this study have been deposited in the Gene Expression Omnibus (GEO) repository under accession number GSE224398 and SRA accession numbers (SRR21079269, SRR21079266, SRR21079265, SRR21079270, SRR21079268, SRR21079267). The results published here are in Fig. 1j, k, l and sup. Figure 5a, b, c obtained from the AD Knowledge Portal (https://adknowledgeportal.org) under study 557 snRNAseqAD_TREM2(syn21126450). Any additional data supporting the findings of this study other than deposited data described previously are available from the lead contact upon request. Source data are provided with this paper.

## Source data

Source data

## Code availability

Code used for single cell RNA-seq data integration and projection, pseudotime analysis is available on a GitHub repository at https://github.com/Jobang9286/APPKI_oligo_scRNAseq.
