# Supplementary material for: Single-cell RNA-sequencing identifies disease-associated oligodendrocytes in male APP NL-G-F and 5XFAD mice
Source: Nat Commun. 2023 Feb 13;14:802. doi: 10.1038/s41467-023-36519-8 (PMC9925742; doi:10.1038/s41467-023-36519-8)
Supplement: Supplementary file 1 — Supplementary information [file 41467_2023_36519_MOESM1_ESM.pdf]

Supplementary Information

Single-cell RNA sequencing identifies disease-associated oligodendrocytes in a mouse model of Alzheimer's disease

Hanseul Park[1#], Byounggook Cho[1#], Hongwon Kim[1#], Takashi Saito[2,3], Takaomi C Saido[2], Kyoung-Jae Won[4 †], Jongpil Kim[1 †]

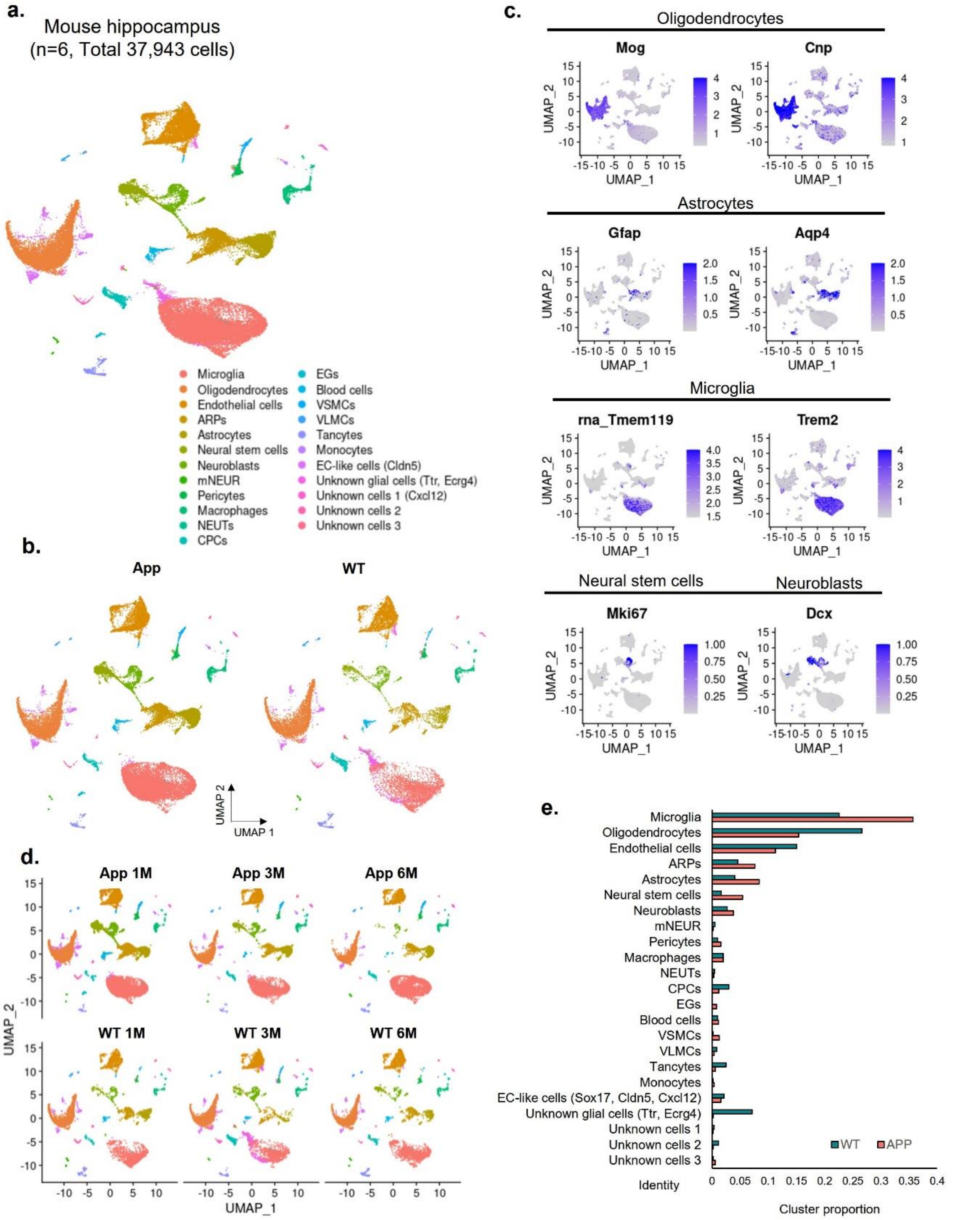

Supplementary Figure 1. Hippocampal scRNA-seq of 1-, 3-, 6-month-old WT and App transgenic mice. (a) UMAP plot showing integrated data in the hippocampal region of WT and App transgenic mice (n=6 animals, a total of 37,943 cells). The entire cell group in the hippocampal region with 23 sub-clusters across time-combined WT and App conditions. (b) UMAP plot showing split hippocampus region clusters by WT and App conditions. (c) UMAP plot showing each cell-specific marker identifying each cluster. (d) UMAP plot showing the entire cell group in the hippocampal region with 23 sub-clusters

along each time-point. (e) Changes in the frequency of entire cell types in App. The ratio of average fraction in age-matched WT versus App transgenic mice. WT: wild-type; M: month.

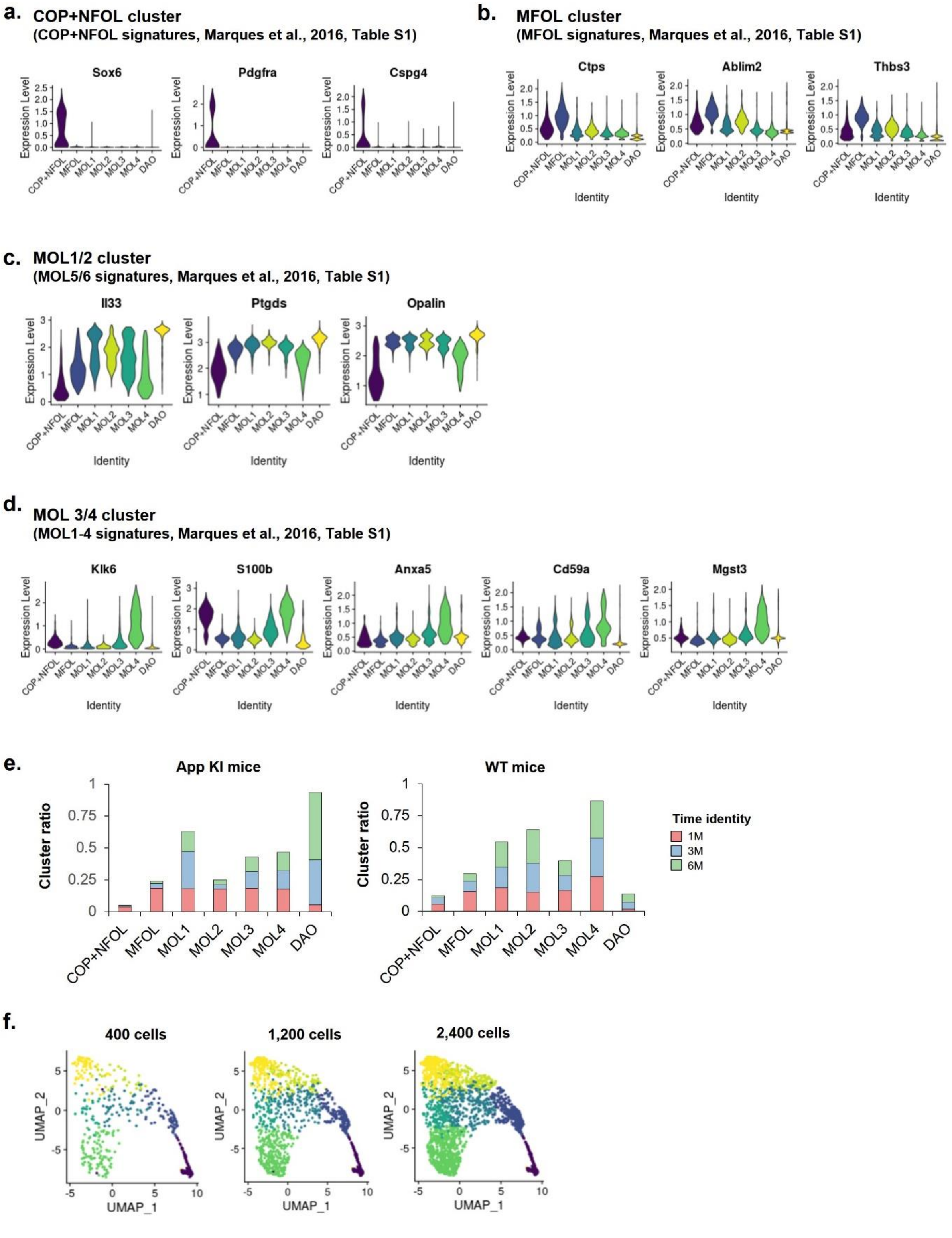

Supplementary Figure 2. App heterogeneity of sub-sorted oligodendrocytes across WT and App conditions. (a-d) Violin plot showing published oligodendrocyte makers as described in Zeisel et. al. 2015, and Marques and Zeisel et. al. 2016. (e) Bar plot showing cluster ratio of oligodendrocytes in APP KI and WT condition mice. (f) UMAP plot showing down-sampled 400, 1200, 2400 oligodendrocytes. WT: wild-type; M: month.

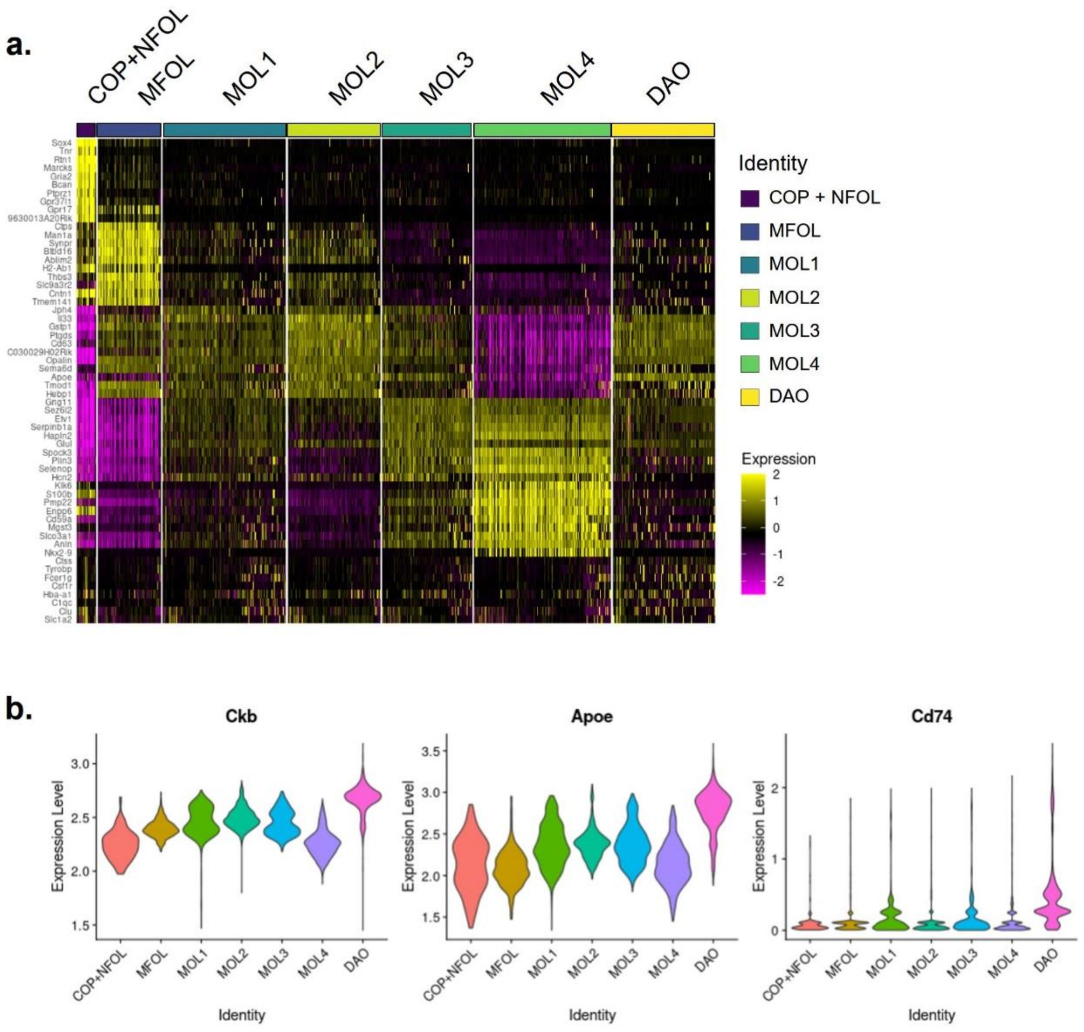

Supplementary Figure 3. Expression profiles of the oligodendrocyte sub-clusters. (a) Heatmap showing top 10 markers of oligodendrocyte sub-clusters. (b) Violin plot showing expression of the immune response genes C4b, Apoe, and Cd74 across oligodendrocyte sub-clusters.

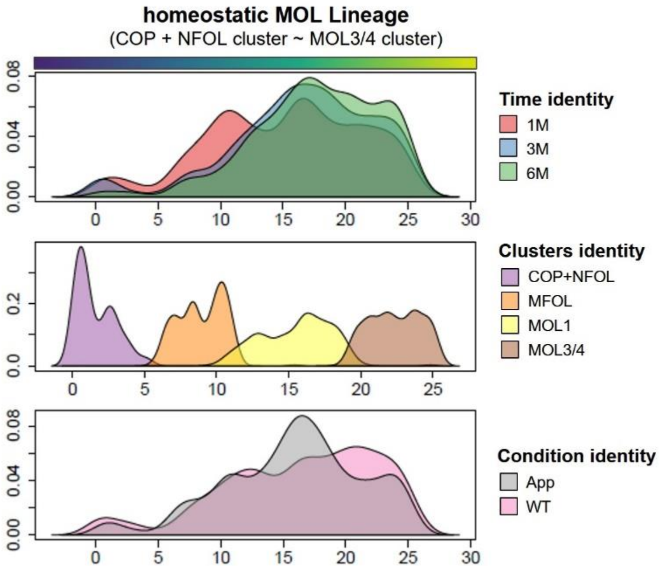

Supplementary Figure 4. Density dynamics of hMOL lineage. Density plot of the continuous trajectory of the hMOL lineage across three-time points, clusters, and conditions. WT: wild-type; M: month.

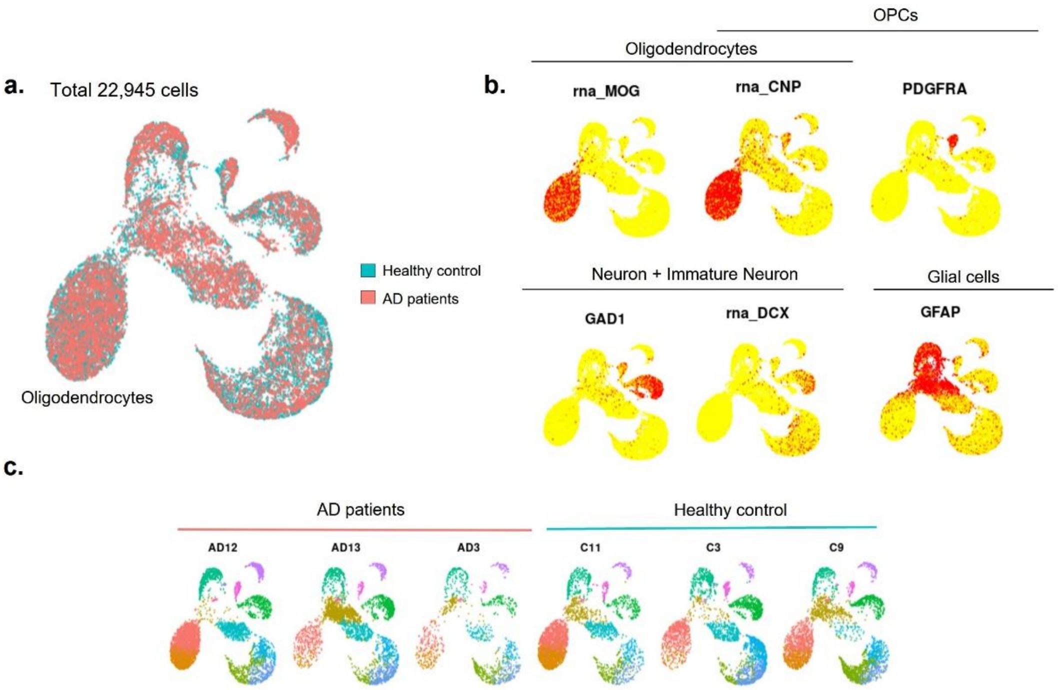

Sample Idenfier	15121461	10298957	20959216	11072071	20280666	20207013
Sample Source	AD_Rush	AD_Rush	AD_Rush	Control	Control	Control
Age	92.443	82.371	97.593	87.953	101.090	96.016
Tissue	Dorsolateral prefrontal cortex					
Cell number	6,068	3,763	1,299	4,050	3,968	3,797
Amyloid	1.956875	8.486875	6.651666667	0	3.559375	0
Sex	Male	Male	Female	Male	Female	Female

Supplementary Figure 5. Public snRNA-seq data of healthy control and postmortem AD. (a) Merged UMAP plot showing all clusters of the dorsolateral prefrontal cortex across each condition (n=6, total 22,945 cells). (b) Each cell-specific marker identifies each cluster across all cell clusters. (c) Information on postmortem AD and healthy controls. AD: Alzheimer’s disease.

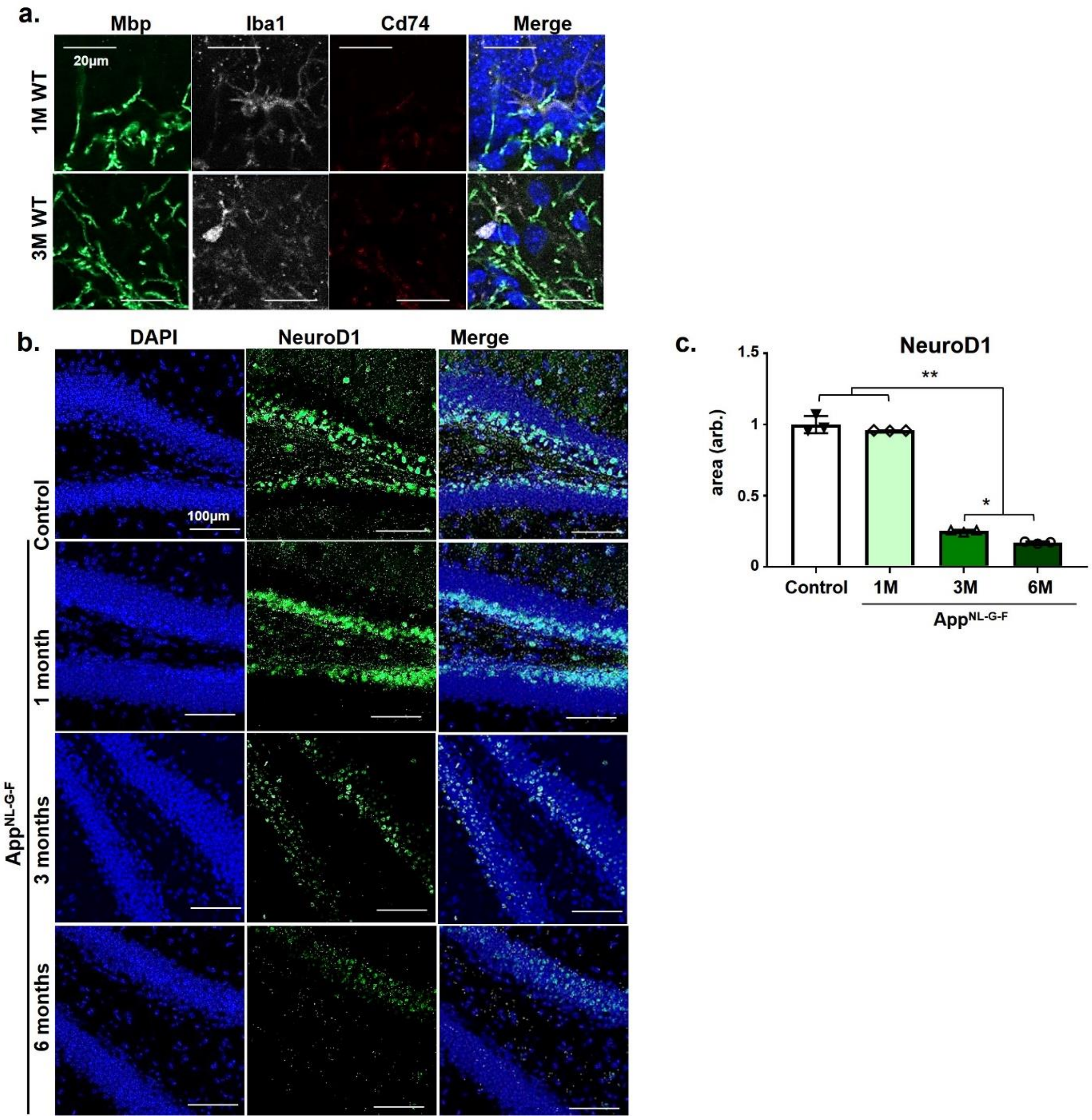

Supplementary Figure 6. Identification of the Cd74+ oligodendrocytes and neuronal cells in WT and AD mice. (a) Immunohistochemistry for Mbp (green), Iba1 (white), Cd74 (red), and DAPI (blue) in the dentate gyrus of WT at 1 and 3 months of age. (b) Immunohistochemistry for NeuroD1 (green) and DAPI (blue) in the dentate gyrus of WT at 6 months of age and App$^{NL\text{-}G\text{-}F}$ mice at 1, 3, and 6 months of age. (c) Area of NeuroD1 cells stained in the DG regions from immunohistochemistry data. Data are expressed as mean ± SEM (n = 3). *p < 0.05, **p < 0.01, one-way ANOVA with Tukey's post hoc test. Source data and p-values are provided as a Source Data file. arb.: arbitrary units.

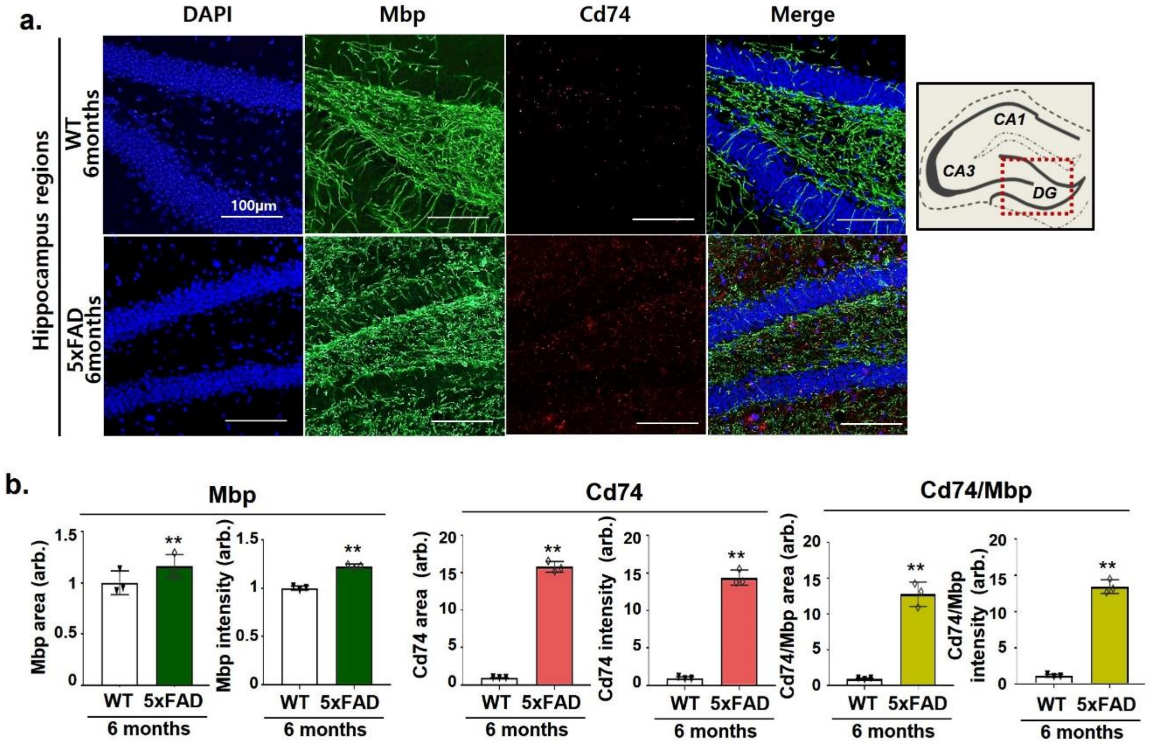

Supplementary Figure 7. 5xFAD mice exhibit DAOs in the hippocampal region (a) Immunohistochemistry for Mbp (green), Cd74 (red), and DAPI (Blue) in the dentate gyrus of control and 5xFAD mice. (b) Area and intensity of Mbp, Cd74, and Cd74/Mbp cells stained in DG regions based on immunohistochemistry data. Data are expressed as mean ± SEM, n = 3. *p < 0.05 and **p < 0.01, two-sided Student's t-test (Mbp: Ctrl vs. 6-month-old 5xFAD area, p = 0.043, Ctrl vs. 6-month-old 5xFAD intensity, p = 0.001; Cd74: Ctrl vs. 6-month-old 5xFAD area, p = 0.001, Ctrl vs. 6-month-old 5xFAD intensity, p = 0.001; Cd74/Mbp: Ctrl vs. 6-month-old 5xFAD area, p = 0.001, Ctrl vs. 6-month-old 5xFAD intensity, p = 0.001). Source data are provided as a Source Data file. arb.: arbitrary units; WT: wild-type.

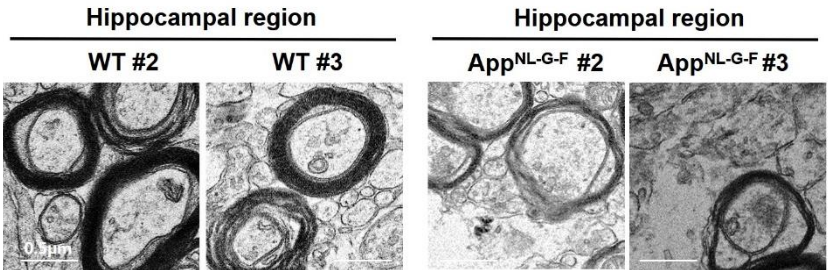

Supplementary Figure 8. Electron micrographs of axons in the DG from 6-month-old WT and AD mice. Two mice per group were used for TEM images. WT: wild-type.

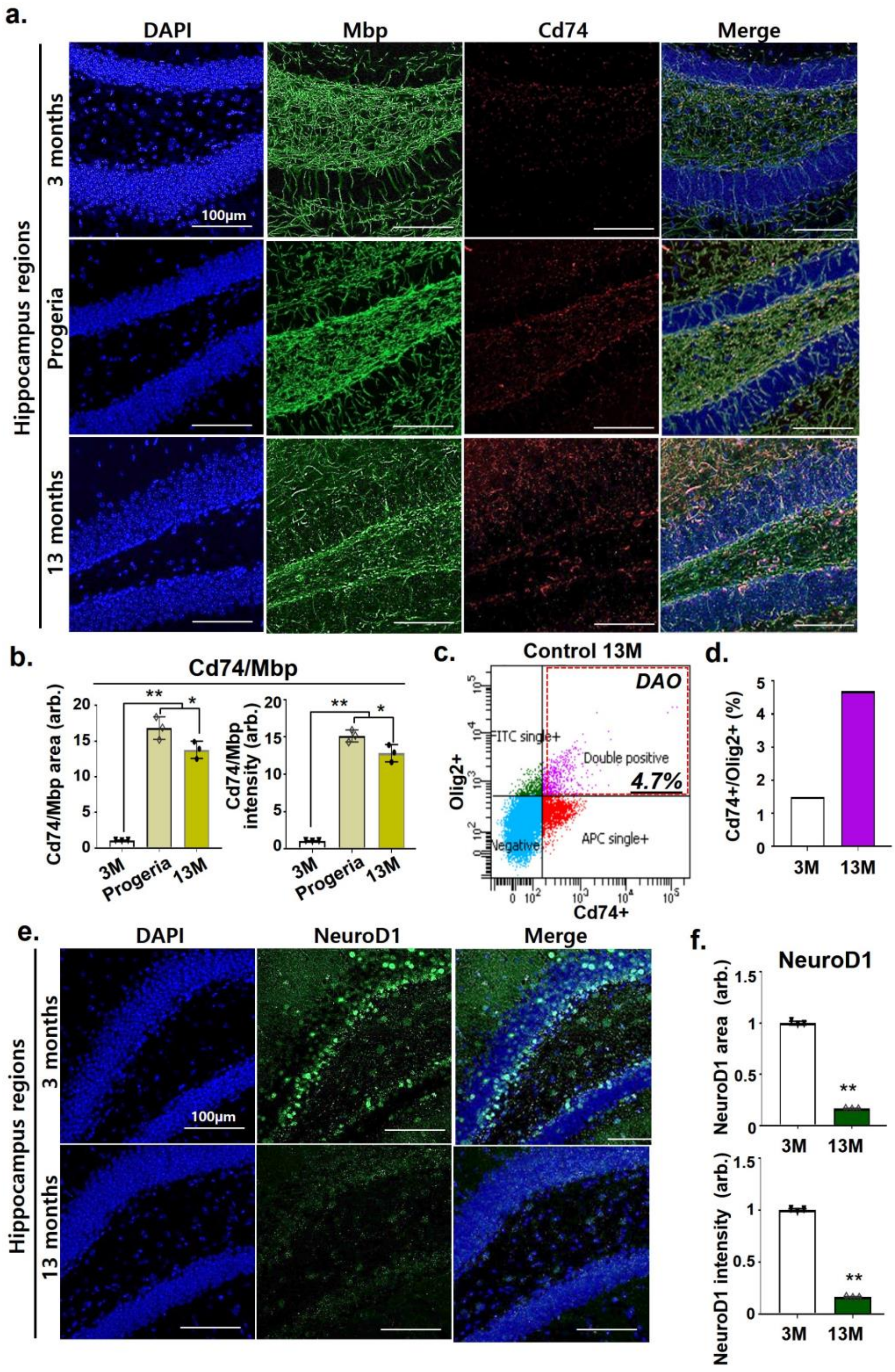

Supplementary Figure 9. DAOs were associated with age. (a) Immunohistochemistry for Mbp (green), Cd74 (red), and DAPI (blue) in the dentate gyrus of 3-month-old, 13-month-old, and Lmna mice. (b) Area and intensity of Cd74/Mbp-positive cells stained in the DG regions from immunohistochemistry data in Supplementary Fig. 8a. Data are expressed as mean ± SEM (n = 3). *$p < 0.05$, and **$p < 0.01$, one-way ANOVA with Tukey's post hoc test. (c) The frequencies of Cd74+ and Olig2+ were determined by flow cytometric analysis. Representative FACS plots are shown. (d) Bar graph illustrating the results

of quantitative FACS analysis of Cd74-positive subsets of the Olig2-positive population. (e) Immunohistochemistry for NeuroD1 (green) and DAPI (blue) in the dentate gyrus of 3- and 13-month-old mice. (f) Area and intensity of NeuroD1 cells stained in the dentate gyrus regions from the immunohistochemistry data in Supplementary Fig. 8e. Data are expressed as mean ± SEM, n = 3. ** $p < 0.01$, two-sided Student's t-test (NeuroD1: Ctrl vs. old area, $p = 0.001$; Ctrl vs. old intensity, $p = 0.001$). Data and *p*-values in b, d, and f are provided as a Source Data file. arb.: arbitrary units; M: month.

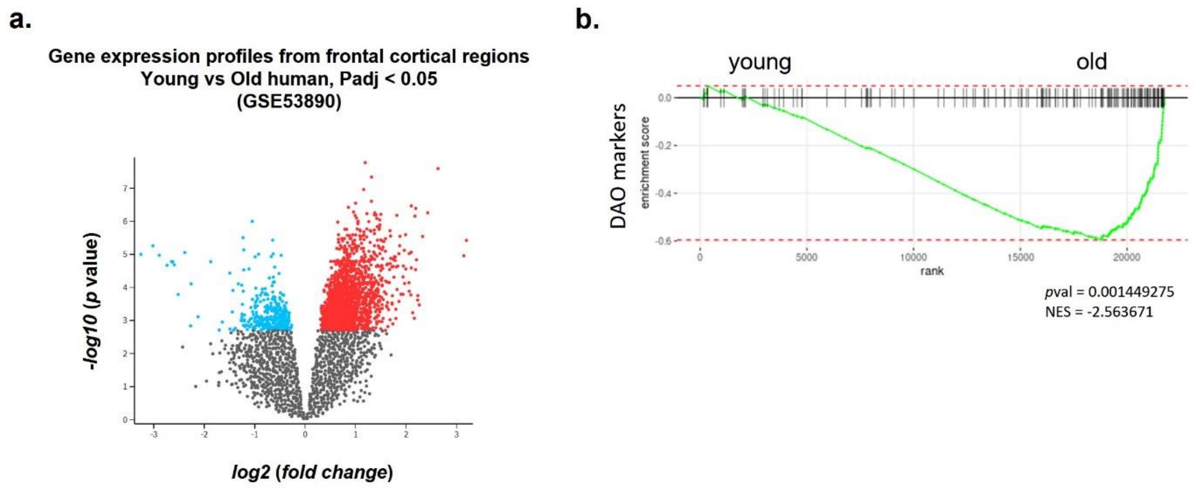

Supplementary Figure 10. Human AD-associated oligodendrocytes from aging and young populations. (a) Volcano plot showing differentially expressed genes across young and old humans; one-sided Fisher's exact test. (b) Gene set enrichment analysis (GSEA) matched DAO markers and young versus old genes; one-sided Fisher's exact test.

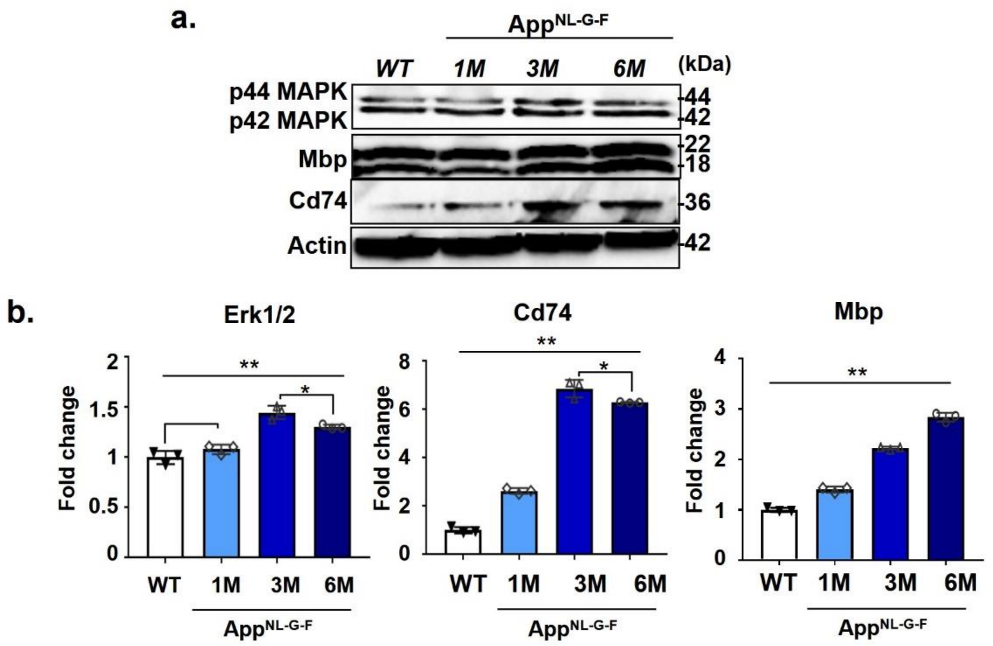

Supplementary Figure 11. Differential gene expression in AD mice according to age. (a) Western blot analysis of Mbp, p44 MAPK/p22 MAPK, Cd74, and Actin in App$^{NL\text{-}G\text{-}F}$ mice. Western blot performed three biological replicates. Source data are provided as a Source Data file. (b) Quantification of western blot results shown in Supplementary Fig. 10a. Data are expressed as mean ± SEM (n = 3). *$p < 0.05$, and **$p < 0.01$, two-way ANOVA with Tukey's post hoc test. Source data are provided as a Source Data file. WT: wild-type; M: month.

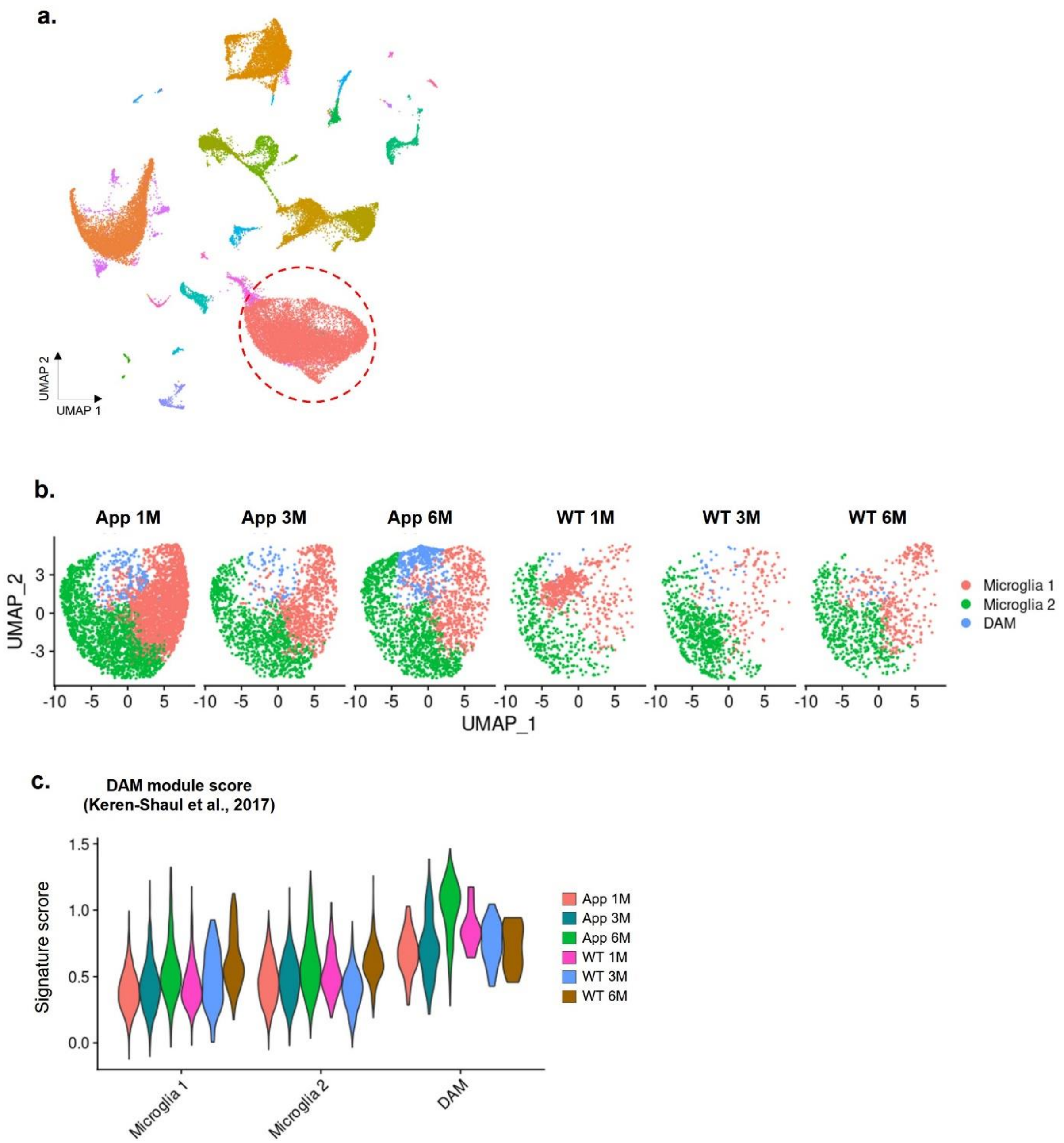

Supplementary Figure 12. Identification of DAM cluster in App transgenic mice. (a) UMAP plot showing entire clusters in the hippocampus region including age-matched WT and App transgenic conditions (red circle; microglia cluster). (b) UMAP plot showing microglia cluster along each time-point and condition. (c) Signature module scores of disease-associated microglia (DAM) enriched in our AD data. Violin plots showing the DAM module score in sub-clusters of WT and App microglia across time-point and condition (calculated from upregulated DAM genes compared to those of homeostatic microglia). WT: wild-type; M: month.

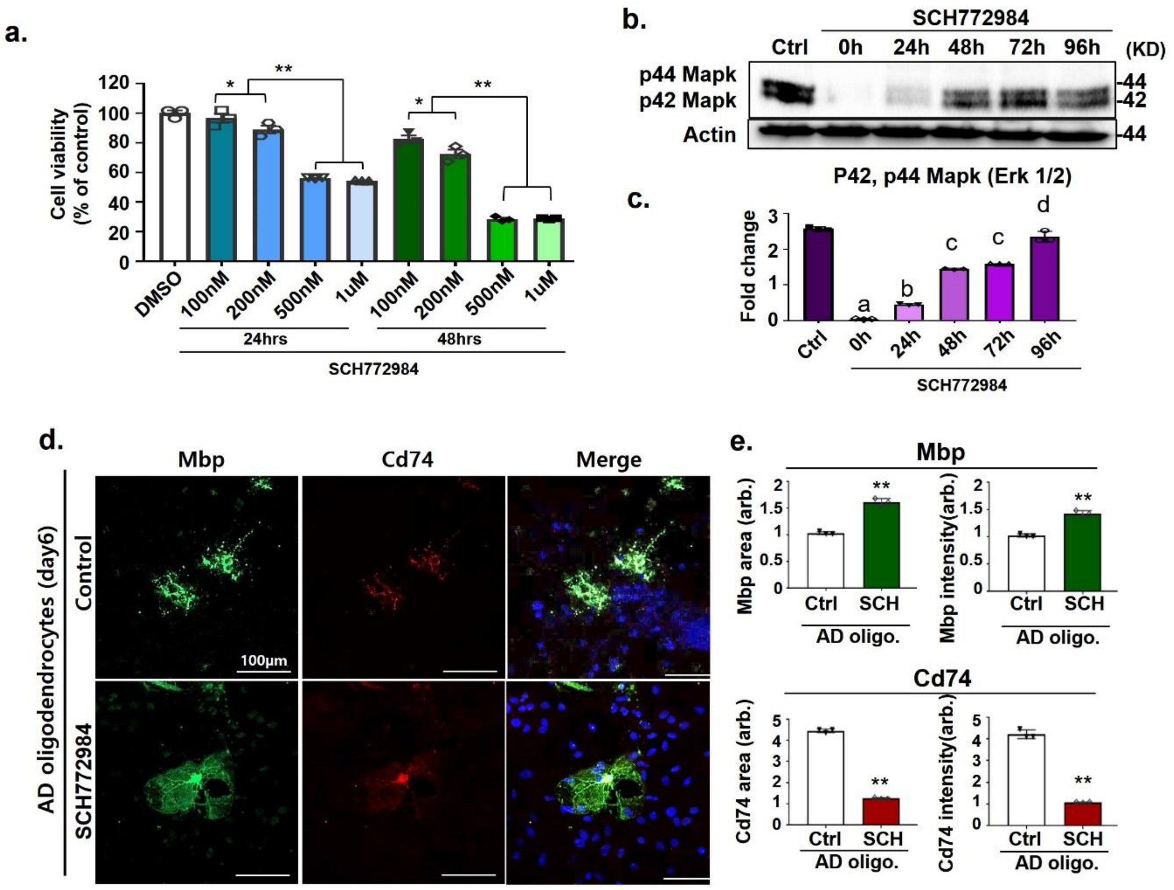

Supplementary Figure 13. Primary culture *in vitro* ERK1/2 inhibitor treatment. (a) Cell viability after 24 and 48 hours of exposure to different concentrations of SCH772984 in primary neurons. Data are expressed as mean ± SEM (*n* = 3). $^{**}p < 0.01$, one-way ANOVA with Tukey's post hoc test. (b) Western blot analysis of p44 MAPK/p22 MAPK in primary neurons according to SCH772984 removal time. Western blot performed three biological replicates. (c) Quantification of western blot results shown in Supplementary Fig. 12b. Data are expressed as mean ± SEM (*n* = 3). Different letters (a, b, c, and d) above the error bar indicate statistical differences, as determined by one-way ANOVA with Tukey's post hoc test ($p < 0.01$). (d) Immunohistochemistry of Mbp (green), Cd74 (red), and DAPI (blue) in App$^{NL\text{-}G\text{-}F}$ primary oligodendrocytes treated with SCH772984 on day six. (e) Quantification of Mbp and Cd74 immunohistochemistry in SCH772984 treated App$^{NL\text{-}G\text{-}F}$ primary oligodendrocytes. Data are expressed as mean ± SEM, n = 3. $^{**}p < 0.01$, two-sided Student's t-test. Exact *p*-values are provided as a Source Data file. Images in b and d are representative of three or more similar experiments. Source data in a, b, c, and e are provided as a Source Data file. arb.: arbitrary units.

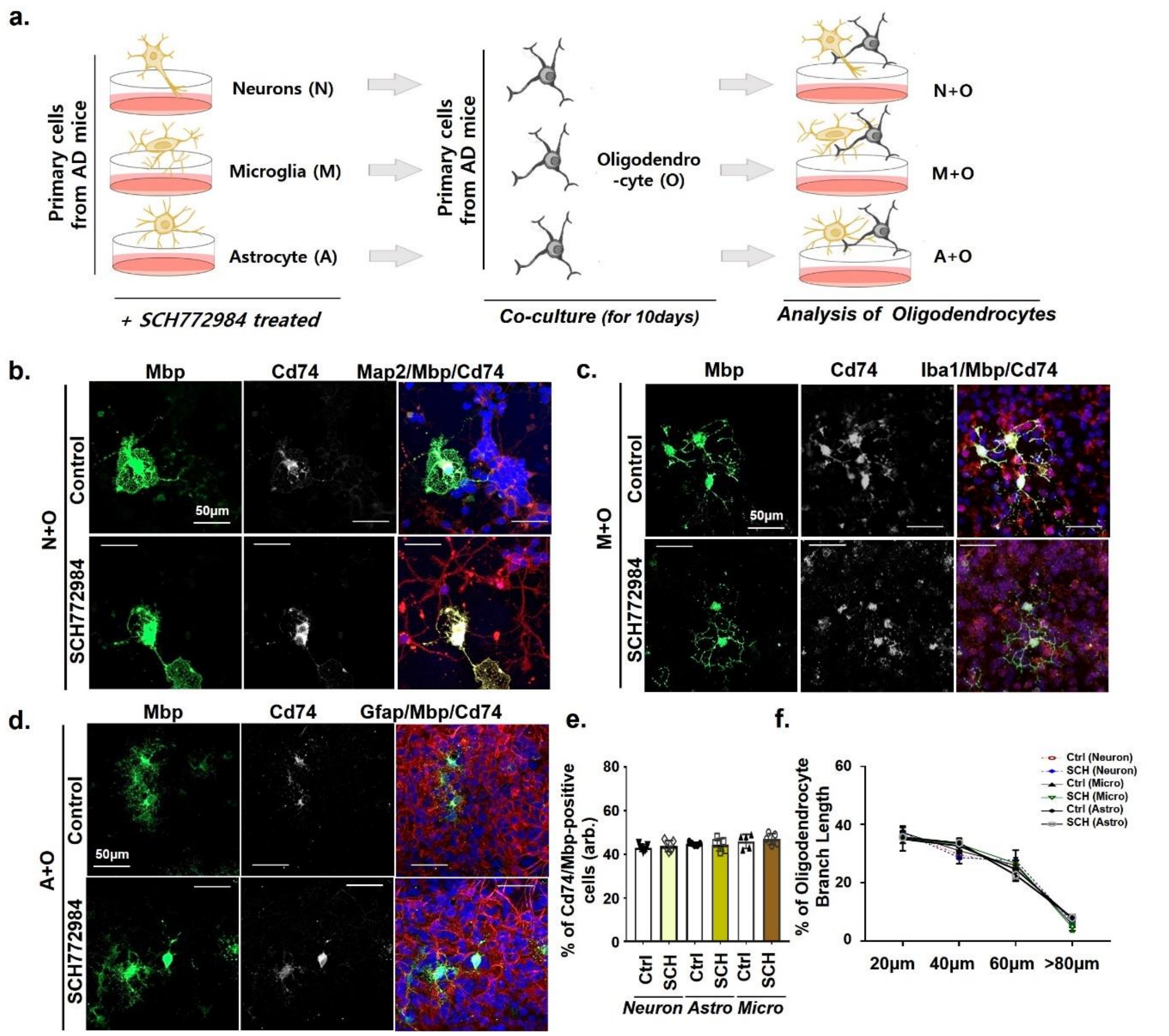

Supplementary Figure 14. Co-cultures of primary neural cells to investigate DAO regulation under ERK-inhibitory conditions. (a) Schematic representation of AD primary neural cell co-culture experimental design. On day one, each neural cell (neurons, microglia, and astrocytes) was isolated by MACS and treated with SCH772984 for seven days. Then, oligodendrocytes were co-cultured in each neural cell. Phenotypes were investigated after 10 days of co-culture. (b) Immunohistochemistry of Mbp (green), Cd74 (white), Map2 (red), and DAPI (blue) in $App^{NL\text{-}G\text{-}F}$ primary neurons and oligodendrocytes treated with SCH772984. (c) Immunohistochemistry of Mbp (green), Cd74 (white), Iba1 (red), and DAPI (blue) in $App^{NL\text{-}G\text{-}F}$ primary microglia and oligodendrocytes treated with SCH772984. (d) Immunohistochemistry of Mbp (green), Cd74 (white), Gfap (red), and DAPI (blue) in $App^{NL\text{-}G\text{-}F}$ primary astrocytes and oligodendrocytes treated with SCH772984. (e) Percentage of Cd74/Mbp-positive cells. Data are expressed as mean ± SEM, n = 3. **p* < 0.05, two-sided Student's t-test (Neuron: Ctrl vs. SCH, *p* = 0.286; Astrocyte: Ctrl vs. SCH, *p* = 0. 386; Microglia: Ctrl vs. SCH, *p* = 0.301). (f) Branch lengths of mouse primary oligodendrocytes after SCH772984 treatment. Data are expressed as mean ± SEM (*n* = 3). **p* < 0.05, ANOVA with Tukey's post hoc test. Source data in e and f are provided as a Source Data file. N: neuron; M: microglia; A: astrocyte; O: oligodendrocyte; Ctrl: control; SCH: sch772984.

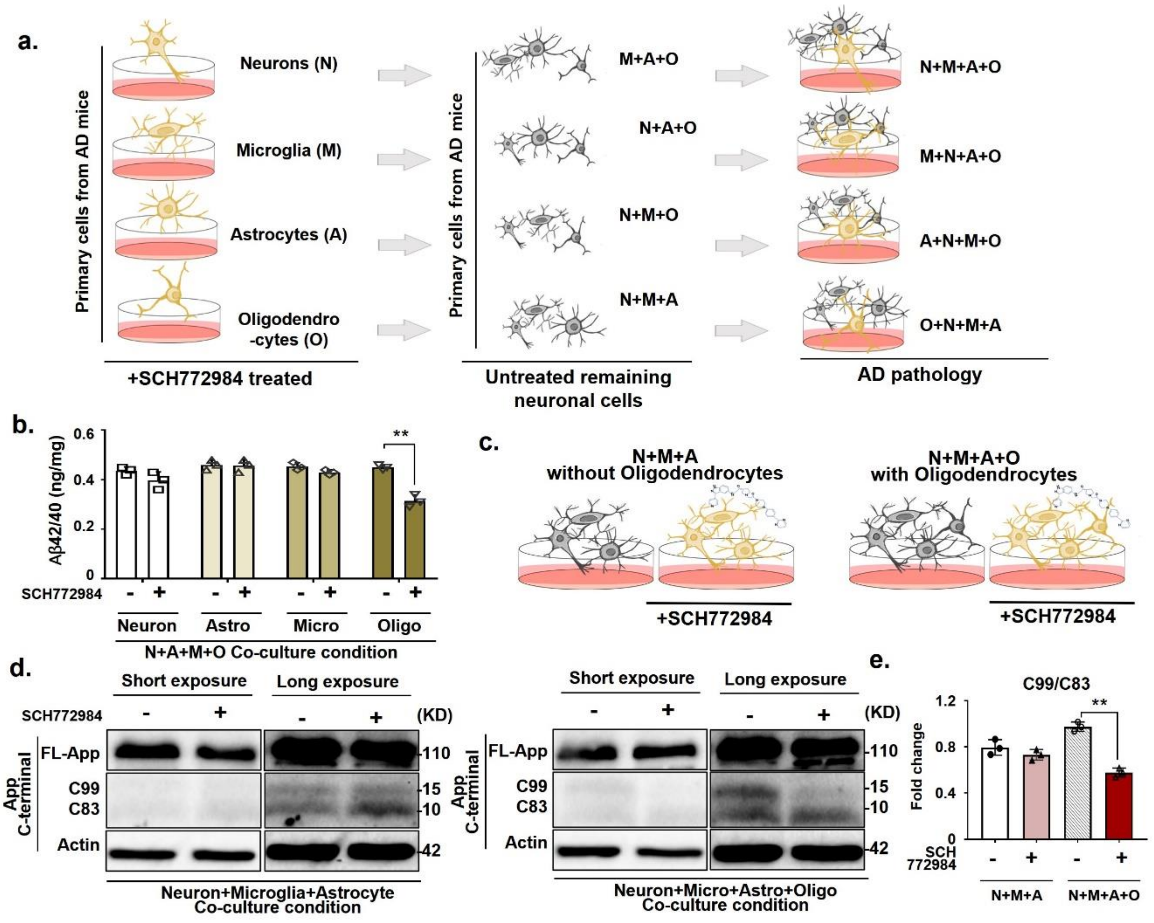

Supplementary Figure 15. Co-cultures of primary neural cells to investigate AD pathogenesis under ERK-inhibitory conditions. (a) Schematic representation of the involvement of Erk1/2 inhibition in AD pathogenesis via SCH772984. On day one, each neural cell (neurons, microglia, astrocytes, and oligodendrocytes) was isolated by MACS and treated with SCH772984 for seven days. Then, triple primary neural cells were co-cultured in each SCH772984-treated neural cell. AD-related phenotypes were investigated after 10 days of co-culture. (b) Aβ42/40 ratio measured by ELISA in SCH772984-treated each of primary neuronal cells. Data are expressed as mean ± SEM, n = 3. **p < 0.01, two-sided Student's t-test (Neuron: Ctrl vs. SCH, p = 0.144; Astrocyte: Ctrl vs. SCH, p = 0.866; Microglia: Ctrl vs. SCH, p = 0.091; Oligodendrocyte: Ctrl vs. SCH, p = 0.001). (c) Schematic representation of the triple and quadruple co-culture conditions. (d) Western blotting of App CTFs and actin in SCH772984-treated primary neuronal co-culture cells. Western blot performed three biological replicates. (e) Quantification of C99/C83 western blot in supplementary Fig. 14d. Data are expressed as mean ± SEM, n = 3. **p < 0.01, two-sided Student's t-test (triple-culture: Ctrl vs. SCH, p = 0.12; quadruple-culture: Ctrl vs. SCH, p = 0.001). Source data in b, d, and e are provided as a Source Data file. N: neuron; M: microglia; A: astrocyte; O: oligodendrocyte.

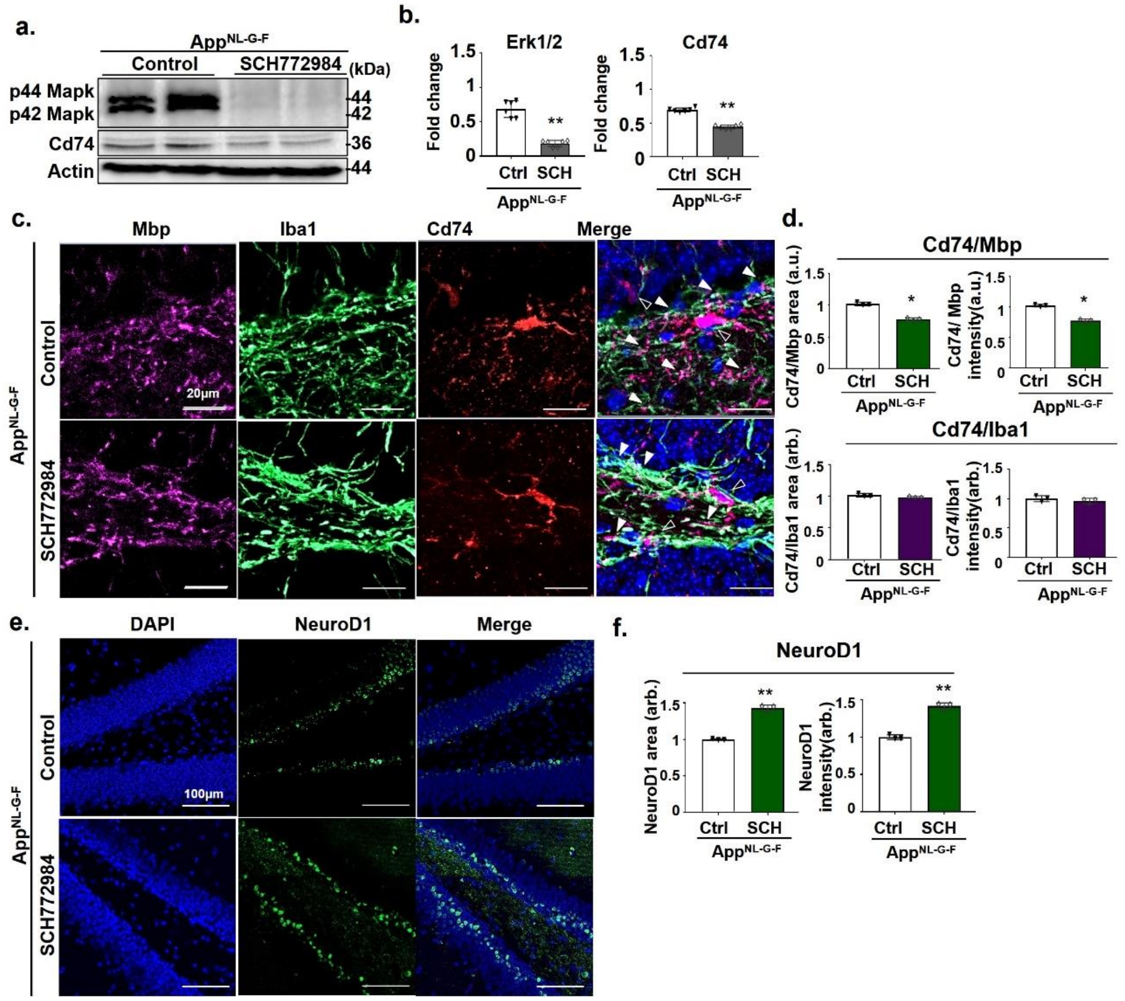

Supplementary Figure 16. ERK1/2 inhibitor treatment rescues AD phenotypes. (a) Western blot analysis of p44/p22 MAPK, Cd74, and Actin in App$^{NL\text{-}G\text{-}F}$ mice with SCH772984 treatment. (b) Quantification of western blot results shown in Supplementary Fig. 15a. Data are expressed as mean ± SEM, n = 3. **p < 0.01, two-sided Student's t-test (Erk1/2, p = 0.001; Cd74, p = 0.001). (c) Immunohistochemistry for Mbp (purple), Iba1 (green), Cd74 (red), and DAPI (Blue) in the dentate gyrus of App$^{NL\text{-}G\text{-}F}$ mice with SCH772984 treatment. (d) Area and intensity of Cd74/Iba1 and Cd74/Mbp-positive cells stained in the DG regions from the immunohistochemistry data in Supplementary Fig. 15c. Data are expressed as mean ± SEM, n = 3. *p < 0.05, two-sided Student's t-test (Cd74/Mbp: Ctrl vs. SCH area, p = 0.001; Ctrl vs. SCH intensity, p = 0.001; Cd74/Iba1: Ctrl vs. SCH area, p = 0.169; Ctrl vs. SCH intensity, p = 0.172). (e) Immunohistochemistry for NeuroD1 (green) and DAPI (blue) in the dentate gyrus of App$^{NL\text{-}G\text{-}F}$ mice with SCH772984 treatment. (f) Area and intensity of NeuroD1 cells stained in the dentate gyrus regions from immunohistochemistry data in Supplementary Fig. 15e. Data are expressed as mean ± SEM, n = 3. **p < 0.01, two-sided Student's t-test (NeuroD1: Ctrl vs. SCH area, p = 0.002; Ctrl vs. SCH intensity, p = 0.001). Source data in a, b, d, and f are provided as a Source Data file. Ctrl: control; SCH: sch772984; arb.: arbitrary units.
