# Supplementary material for: Single-cell RNA-sequencing identifies disease-associated oligodendrocytes in male APP NL-G-F and 5XFAD mice
Source: Nat Commun. 2023 Feb 13;14:802. doi: 10.1038/s41467-023-36519-8 (PMC9925742; doi:10.1038/s41467-023-36519-8)
Supplement: Supplementary file 2 — Description of Additional Supplementary Files [file 41467_2023_36519_MOESM2_ESM.pdf]

Description of Additional Supplementary Files

File Name: Supplementary Data 1

Description: Differentially expressed genes in oligodendrocyte cell states.

File Name: Supplementary Data 2

Description: Transcriptional signatures in DAO versus other clusters.

File Name: Supplementary Data 3

Description: List of samples used in scRNA-seq analysis
